# Poplar leaf bud resin metabolomics: seasonal profiling of leaf bud chemistry in *Populus trichocarpa* provides insight into resin biosynthesis

**DOI:** 10.1093/pcp/pcae149

**Published:** 2024-12-19

**Authors:** Eerik-Mikael Piirtola, David P Overy, C. Peter Constabel

**Affiliations:** Centre for Forest Biology, Department of Biology, University of Victoria, 3800 Finnerty Road, Victoria, British Columbia V8P 5C2, Canada; Ottawa Research and Development Centre, Agriculture and Agri-Food Canada, 960 Carling Avenue, Ottawa, Ontario K1A 0C6, Canada; Centre for Forest Biology, Department of Biology, University of Victoria, 3800 Finnerty Road, Victoria, British Columbia V8P 5C2, Canada

**Keywords:** poplar, bud resin, dihydrochalcone, metabolomics, phenylpropanoid, flavonoid, methylation

## Abstract

**Trees in the genus *Populus* synthesize sticky and fragrant resins to protect dormant leaf buds during winter. These resins contain diverse phenolic metabolites, in particular, hydroxycinnamate esters and methylated flavonoids. *Populus trichocarpa* leaf bud resin is characterized by methylated dihydrochalcone aglycones. To determine how the resin profile is influenced by seasonal changes, *P. trichocarpa* lateral leaf bud extracts and secreted surface resin were collected monthly over a 1-year cycle. The dihydrochalcones in both sets of extracts were quantified using ultrahigh pressure liquid chromatography–mass spectrometry (UPLC–MS), and other chemical changes were monitored using nontargeted metabolomics by UPLC–high-resolution MS (UPLC–HRMS). The results indicate that the dihydrochalcone content changes over the seasons and that biosynthesis occurs concomitant with bud development in the summer months. Nontargeted metabolomics data confirmed a pattern of dramatic changes in the summer and further suggested additional periods of substantive biochemical change in the resin. While overall patterns of surface-extracted resin matched those of whole bud extracts, some of the dynamics were shifted in the surface resin samples. This study provides the basis for the use of dihydrochalcones and other identified resin components as metabolic markers for more detailed investigations of resin biosynthesis, secretion, and movement to the bud surface**.

## Introduction

The genus *Populus* comprises widely distributed temperate trees native to North America, Europe, and Asia (Mitton and Grant 1996, Cooke and Rood 2007). It includes poplars, aspens, and cottonwoods; here, all are referred to as poplars. They play significant ecological roles and can act as keystone species or have economical importance in rapid rotation forestry or reforestation. *Populus* has become a model for tree molecular biology and genomics, in part as several *Populus* hybrids can be genetically transformed (Jansson, and Douglas,, 2007). *Populus* species produce a diverse suite of secondary metabolites and are rich in phenolic, phenylpropanoid, and flavonoid compounds. Many of these phytochemicals have demonstrated roles in plant defense and stress adaptation (Constabel et al. 2014), and *Populus* has become an important system for understanding the functions and chemical ecology of phenolic secondary metabolites in plants (Constabel and Lindroth 2010; Tsai et al. 2006). Furthermore, poplars have long-standing uses in traditional medicine due to their analgesic and anti-inflammatory properties (Adams et al. 2009, Uprety et al. 2012).

Poplar leaves, roots, and bark contain abundant phenylpropanoid and phenolic compounds, including condensed tannins, salicinoids, and hydroxycinnamic acids (Constabel and Lindroth 2010). Condensed tannins are mixtures of oligomeric and polymeric flavan-3-ols and can act as antimicrobials and cellular antioxidants, as well as deter some types of herbivores (Barbehenn and Constabel 2011, Gourlay et al. 2022). Salicinoids are complex derivatives of salicyl alcohol glucosides, typically esterified with phenolic acids and are unique to the Salicaceae (poplars and willows). They are abundant in leaves and bark and are act as defenses against herbivory (Boeckler et al. 2011). Hydroxycinnamate derivatives include the widespread caffeoyl quinate (chlorogenic acid) and caffeoyl shikimate esters. Most of these phenolics are water soluble and accumulate intracellularly in the vacuole or are deposited in the cell wall. Poplar leaves have also been found to produce terpenes, benzenoids, and nitrogen-containing compounds as volatiles following herbivory or tissue damage (Irmisch et al. 2014, McCormick et al. 2014).

**Figure 1.**
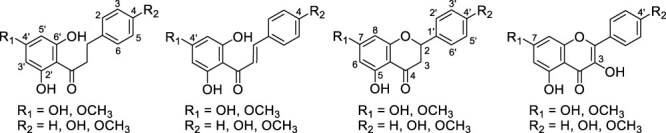
General structures of flavonoids identified in *Populus*: (a) dihydrochalcones, (b) chalcones, (c) flavanones, and (d) flavonols.

In addition, poplars synthesize a variety of lipophilic phenolic and phenylpropanoid compounds as components of the sticky resin found in dormant buds. Leaf bud resin is an adaptation of many temperate trees to protect meristematic tissues and embryonic leaves during the winter months, which coats the leaves as they expand and emerge (Langenheim 2003). Detailed anatomic studies in *Populus deltoides* suggest that the resin is formed on the surface of the leaf bud scales (Curtis and Lersten 1974). The chemical composition of bud resin from many *Populus* species has been extensively characterized by Greenaway and colleagues using gas chromatography–mass spectrometry (GC–MS) (English et al. 1991, 1992, Greenaway et al. 1991a) and more recently using liquid chromatography–MS (LC–MS) methods (Kuś et al. 2018). These studies demonstrate that *Populus* resins comprise mixtures of phenolics, phenylpropanoids and flavonoids; they may also contain alkanes, benzenoids, and terpenoids and other volatiles (Greenaway et al. 1992; Kuś et al. 2018, Pobłocka-Olech et al. 2024). The most common components of poplar bud resin are hydroxycinnamic acids and their esters, which are important for the resin’s characteristic fragrance (Langenheim 2003). Benzoic acid derivatives are also typically found. Nonvolatile resin constituents can include diverse flavonoids: dihydrochalcones, chalcones, flavones, flavanone, flavanols, and flavonols (Fig. 1) are often present (Greenaway and Whatley 1990, English et al. 1991, 1992, Isidorov and Vinogorova 2003). Due to this rich phytochemical content, the resins from several poplar species are valued for their medicinal properties. In the North American *P. balsamifera*, the spring leaf bud resin is abundant and fragrant and widely used in traditional medicine in salves for skin ailments or as wound dressings (Uprety et al. 2012).

Interestingly, the chemical profiles of *Populus* leaf bud resin have been shown to differ dramatically between species (Greenaway et al. 1989, 1992, Greenaway and Whatley 1990, English et al. 1992). For example, *P. deltoides* resin is rich in flavonoids including flavones, flavanols, and chalcones, while *P. nigra* resin is characterized by high hydroxycinnamate ester content (Greenaway et al. 1992; Kuś et al. 2018). By contrast, *P. alba* resin contains almost no flavonoids, but rather accumulates hydrocarbons such as heptacosane (Greenaway et al. 1992). Unlike their intracellular counterparts, resin flavonoids are found as aglycones, which reduces their water solubility (Greenaway and Whatley 1990, Greenaway, et al., 1991b). In addition, resin flavonoids are typically functionalized by the presence of one or more *O*-methylations (Wollenweber 1974, English et al. 1991), further contributing to the hydrophobic nature of the resin. Flavonoid aglycones and *O*-methylated flavonoids are also found in secreted exudates of other trees such as birch (*Betula*) and alder (*Alnus*) (Wollenweber and Dietz 1981, Valkama et al. 2004).

Resins from *P. trichocarpa* (black cottonwood) and the closely related *P. balsamifera* are unique in containing abundant dihydrochalcones (English et al. 1991). In addition, the resin is rich in benzenoids such as benzyl benzoate, benzyl salicylate, and 4-acetophenone, as well as hydroxycinnamate esters such as coumaryl cinnamate and cinnamyl cinnamate (English et al. 1991). Other flavonoids reported include several methylated chalcones and flavanones, common resin components, in addition to dihydrochalcones. However, the latter compounds are by far the most abundant flavonoid constituents of the bud resin of this species (English et al. 1991). Dihydrochalcones constitute a relatively small class of flavonoids with approximately 256 identified structures (Rivière 2016), but they can be found in over 46 plant families. They are closely related to chalcones, common flavonoid intermediates, and are formed via the reduction of the double bond in the three-carbon bridge of chalcones (Fig. 1). The double bond reductase has been investigated, but its identity is not entirely resolved (Ibdah et al. 2018). Well-known dihydrochalcones include phloretin and its glycosylated form, phloridzin, which accumulate to high levels in apple leaves and fruit (Ibdah et al. 2018). These compounds are thought to be important for antifungal defense and are also proposed to contribute to human health.

Dihydrochalcone aglycones with varying degrees of *O*-methylation and hydroxylation substitution patterns were previously reported in leaf bud extracts from both *P. trichocarpa* and *P. balsamifera* (Greenaway and Whatley 1990, English et al. 1991, Greenaway et al. 1992). Strikingly, these compounds are largely absent in the leaf bud resin of most other poplars (Greenaway et al. 1991a, 1992, English et al. 1992). However, other *O*-methylated dihydrochalcones are found in plant extracts of other plant species, such as *Piper aduncum* (Orjala et al. 1994) and *P. dennisii* (Cabanillas et al. 2012), both of which have been shown to possess medicinal significance (Holdsworth and Damas 1986, Céline et al. 2009).

The chemical diversity of poplar leaf bud resins has been previously characterized, but not in a temporal context. Observational evidence suggests that extrusion of resin to the bud surface is stimulated in the spring, but resin is already present in late summer and fall. Thus, the timing of resin compound biosynthesis and whether there are seasonal shifts in its composition are not known. These questions motivated us to study the accumulation and the potential changes in chemical composition of leaf bud resin in *P. trichocarpa* over a 1-year cycle. We focused on this species’ abundant and characteristic flavonoids, the dihydrochalcones, using a targeted approach. In parallel, we studied the same samples in a nontargeted metabolomics method using high-resolution MS (HRMS) in order to investigate seasonal changes on a broad scale. We applied these methods to both whole bud extracts and surface resin. Our work shows a clear pattern of resin

synthesis in the summer and stable bud resin composition in the winter. It also illustrates clear differences in the seasonal dynamic of extruded surface resin and whole bud resin.

## Results

### Dihydrochalcone accumulation within leaf buds is linked to bud growth and development

**Figure 2.**
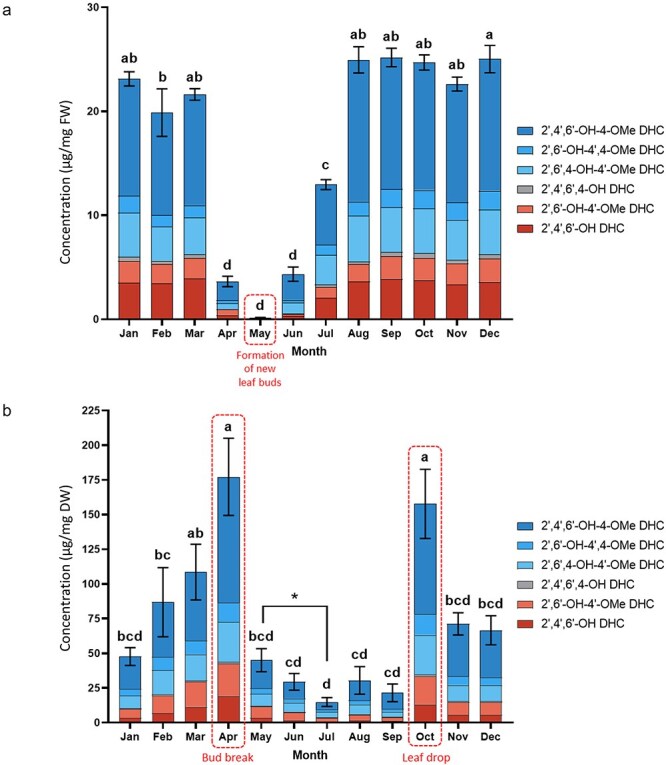
Quantification of the total amount of dihydrochalcones in poplar bud resin extracts. Quantification of dihydrochalcones (DHCs) in (a) whole leaf buds normalized by fresh weight and (b) secreted surface resin normalized by dry extract weight. The data display means ± cumulative SE for whole leaf buds (*n* = 3) and surface resin (*n* = 9). Quantified analytes include 2ʹ,4ʹ,6ʹ-4ʹ-OMe DHC (2ʹ,4ʹ,6ʹ-trihydroxy-4-methoxydihydrochalcone), 2ʹ,6ʹ-OH-4,4ʹ-OMe DHC (2ʹ,6ʹ-dihydroxy-4,4ʹ-dimethoxydihydrochalcone), asebogenin (2ʹ,6ʹ,4-OH-4ʹ-OMe DHC), 2ʹ,4ʹ,6ʹ,4-OH DHC (phloretin), 2ʹ,6ʹ-4ʹ-OMe DHC (2ʹ,6ʹ-dihydroxy-4ʹ-methoxydihydrochalcone), and 2ʹ,4ʹ,6ʹ-OH DHC (2ʹ,4ʹ,6ʹ-trihydroxydihydrochalcone). Significant differences (*P* < .05) between the sampling months are indicated by different letters as determined by Tukey’s *post hoc* HSD. Major phenological stages of the leaf bud development are annotated and indicated by dashed lines. May corresponds to the first stage of the leaf bud development, while April corresponds to bud break. October coincides with leaf drop. Surface resin samples collected between May and July indicated by * included the whole leaf nodes, including the leaf primordia and the flushed leaves. Quantification of the individual compounds is presented in Figs S2 and 3.

In order to study the seasonal dynamics of *P. trichocarpa* leaf bud resin, we harvested whole leaf buds and collected surface resin samples monthly in a seasonal time course with replicated clonal trees grown in a common garden. We first analyzed these samples for dihydrochalcone content and composition. Both whole leaf bud extracts (normalized by tissue fresh weight; Supplementary Fig. S2) and surface resin methanol washes (normalized by extract dry weight; Supplementary Fig. S3) were collected. In whole leaf bud extracts, dihydrochalcone content was consistently high (20–25 µg/mg bud fresh weight) from August to March (Fig. 2a). A sharp reduction in dihydrochalcones was observed in April and May, coinciding with bud break, rapid expansion of leaves from the buds, and the formation of new lateral buds in the axils of new leaves. From June to August, there was a strong increase in dihydrochalcone accumulation as the new buds develop. The relative proportions of dihydrochalcone constituents remained constant throughout the year. For example, 2ʹ,4ʹ,6ʹ-OH-4-OMe dihydroxychalcone (DHC) was the most abundant of the dihydrochalcones detected at all time points, whereas 2ʹ,4ʹ,6ʹ,4-OH DHC, the precursor for *O*-methylated dihydrochalcones, was consistently detected at the lowest concentration.

Dihydrochalcone content in the surface resin over time was more dynamic compared to the whole leaf bud extracts (Fig. 2b). Consistent with our analysis of whole leaf bud extracts, 2ʹ,4ʹ,6ʹ-OH-4-OMe DHC was observed as the most abundant of the dihydrochalcones and 2ʹ,4ʹ,6ʹ,4-OH DHC was observed as the least abundant dihydrochalcone in the secreted surface resin. In these extracts, dihydrochalcones were consistently present from October to April, with concentrations between 50 and 100 µg/mg dry weight. They also showed a sharp drop in concentration during bud development in the summer months, but spiked in April and October (Fig. 2b). Because the surface resin samples were normalized by extract dry weight, the dihydrochalcone concentrations in these extracts would have been more impacted by changes in other resin components than the whole bud extracts (Fig. 2a).

### Nontargeted metabolomics analysis in *P. trichocarpa* whole leaf bud extracts reveals seasonal patterns

**Figure 3.**
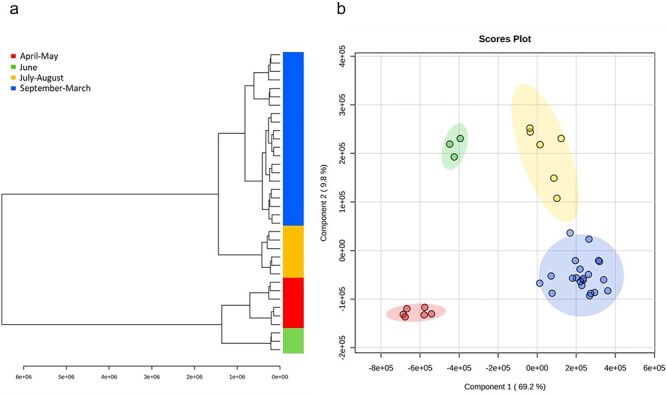
Nontargeted metabolomics analysis of *P. trichocarpa* whole leaf bud extracts. (a) Hierarchical cluster dendrogram and (b) PLS-DA score plot of *P. trichocarpa* whole leaf bud extract metabolites detected in positive ionization mode. Samples clustered together are identified by color coding and named based on their sampling months. Multivariate analysis was conducted using MetaboAnalyst 5.0 (Pang et al., 2021). The highlighted area around the data points signifies the 95% confidence regions.

Nontargeted metabolomic profiling was performed to further investigate the effects of the seasons on the chemical composition of the *P. trichocarpa* whole leaf buds. Metabolomic data processing of the ultrahigh pressure LC (UPLC)–HRMS profiles yielded a data matrix of 298 mass features, expressed as an amalgamation of retention time and mass to charge ratio (RT_*m/z*). Hierarchical cluster analysis was performed to inspect the sample grouping of the extracts based on similarities in the mass feature profiles (Fig. 3a). Four distinct clusters of samples were observed: April–May, June, July–August, and September–March. Partial least squares discriminate analysis (PLS-DA) of the data matrix yielded a similar separation between these four sample groups explaining a cumulative 79% of the variability in the data model within the first two components (Fig. 3b; the obtained variance scree plot and loading plot for the PLS-DA analysis are shown in Supplementary Fig. S4). The sample grouping appeared to follow a linear pattern where the groups consisted of subsequent sampling time points. The April–May cluster coincided with the initiation of bud break and the emergence of new leaf buds. The April time point comprised fully opened buds and emerging leaves, while in May, extracts were from newly formed leaf primordia. June samples formed their own cluster, representing an early stage in the development of leaf buds. The July–August cluster coincided with the active growth and development of leaf buds. The largest cluster spanned September to March, aligning with dormancy and overwintering of the leaf buds.

A qualitative differential analysis was conducted to explore the distribution of mass features among the four sample groups (Supplementary Fig. S5). Of the 298 detected mass features, 155 were common to all four sample groups. The July–August group had the highest metabolite diversity with 272 detected mass features, whereas the April–May group had the lowest diversity, with 186 detected mass features. Notably, samples collected in April and May contained the highest number of unique mass features, followed by June, September–March, and July–August groups. In pairwise comparisons, the July–August and September–March groups showed the most overlap, while September–March and June groups, as well as September–March and April–May groups, shared only one common unique mass feature. Overall, this analysis suggests that many mass features were consistently present across the identified sample groups and that the spring samples were the most differentiated from other groups.

**Figure 4.**
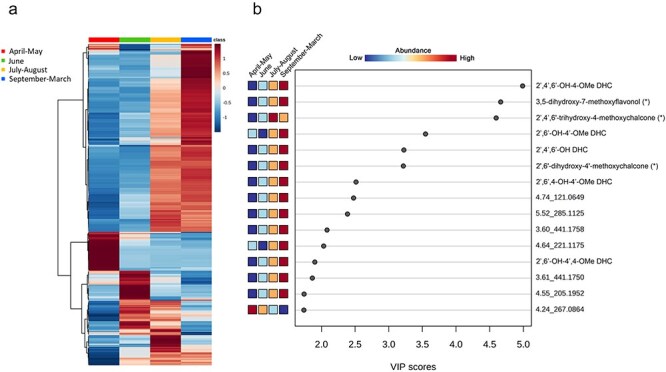
Metabolite distribution analysis of *P. trichocarpa* whole leaf bud extracts. (a) Hierarchical cluster heatmap of mass feature distribution representing the relative abundance of 298 mass features between the different sample groups. Sample groups are indicated by color coding. (b) VIP scores for detected mass features. A higher VIP score signifies a higher importance in Component 1 of PLS-DA. The colored boxes indicate the relative abundance of a mass feature between the sample groups. Where available, the mass features are annotated based on matching *m*/*z* of [M + H]^+^ and RT of analytical standards, including 2ʹ,4ʹ,6ʹ-OH-4-OMe DHC (2ʹ,4ʹ,6ʹ-trihydroxy-4-methoxydihydrochalcone), 2ʹ,6ʹ-OH-4ʹ-OMe DHC (2ʹ,6ʹ-dihydroxy-4ʹ-methoxydihydrochalcone), 2ʹ,4ʹ,6ʹ-OH DHC (2ʹ,4ʹ,6ʹ-trihydroxydihydrochalcone), 2ʹ,6ʹ,4-OH-4ʹ-OMe DHC (asebogenin), and 2ʹ,6ʹ-OH-4,4ʹ-Me DHC (2ʹ,6ʹ-dihydroxy-4,4ʹ-dimethoxydihydrochalcone). Putatively identified mass features are indicated by *. Naming includes RT followed by *m*/*z* for the unidentified mass features.

Hierarchical cluster analysis of the mass features was conducted to explore the distribution of metabolites within the four sample groups based on relative abundances (Fig. 4a). In parallel, from the PLS-DA data model, mass features with the highest variable importance in projection (VIP) scores for Component 1 (Fig. 4b) and Component 2 (Supplementary Table S1) were compared. Mass features with a high VIP score are those that significantly contribute to the separation between the sample groups. In this analysis, among the 15 highest-scoring mass features, we identified dihydrochalcones and putative *O*-methylated flavonoids (Fig. 4b). Notably, the highest VIP score was attributed to 2ʹ,4ʹ,6ʹ-OH-4-OMe DHC, which was the most prevalent dihydrochalcone in the targeted analysis of the whole leaf bud extracts. *O*-Methylated dihydrochalcones with high VIP scores were found to be abundant in the September–March group. In addition to dihydrochalcones, we putatively identified the second highest-scoring mass feature 4.80_285.0762 as 3,7-dihydroxy-5-methoxyflavonol, previously reported as a component of bud resin in *P. balsamifera* (Greenaway and Whatley 1990). The mass feature with the highest VIP score for the July–August group was 4.72_287.0915, which could be tentatively identified as 2ʹ,4ʹ,6ʹ-trihydroxy-4-methoxychalcone based on the accurate mass. The identity of this mass feature could not be confirmed due to the lack of an analytical standard. However, this chalcone had been previously reported in *P. trichocarpa* leaf buds (English et al. 1991). Another unidentified mass feature 4.24_267.0864 achieved the highest VIP score out of the mass features with high abundance in the April–May group. The top 15 mass features with the highest VIP scores were largely identical between Component 1 and Component 2.

In total, 63 mass features with a VIP score of ≥1 (Component 1) are cataloged in Supplementary Table S2. In addition to flavonoids, we identified the salicinoids, salicortin and tremulacin, as well as benzyl benzoate, in the whole leaf buds. These compounds have been previously reported in *P. trichocarpa* (English et al. 1991, Fellenberg et al. 2020).

### *Populus trichocarpa* leaf bud surface resin displays seasonal metabolite patterns distinct from whole bud extracts

**Figure 5.**
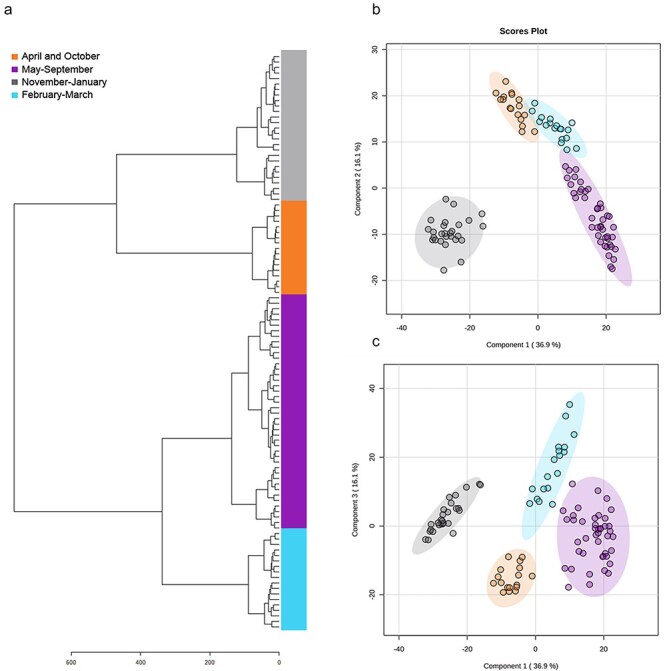
Nontargeted metabolomics analysis of *P. trichocarpa* leaf bud secreted surface resin extracts. (a) Hierarchical cluster dendrogram and PLS-DA scores plots for (b) Components 1 and 2 and (c) Components 1 and 3. *Populus trichocarpa* surface resin extract metabolites were analyzed in positive ionization mode. Grouped samples are identified by color coding and named based on their sampling months. Multivariate analysis was conducted using MetaboAnalyst 5.0 (Pang et al., 2021). Highlighting around the data points signifies the 95% confidence regions.

The same UPLC–HRMS nontargeted metabolomics methods and workflows were applied to the surface resin extracts. This generated a data matrix of 330 mass features. Hierarchical cluster analysis and PLS-DA data modeling again grouped the samples into four distinct sample groups based on the variance in mass feature profiles. These clusters grouped samples from November to January, February to March, April and October, and May to September (Fig. 5a). These groupings only partially reflected the dihydrochalcone profiles across the seasons and were distinct from the seasonal groupings for whole leaf bud extracts (Fig. 3). Interestingly, April and October clustered together, setting them apart from the other sample groups which predominantly consisted of consecutive sampling points. PLS-DA analysis confirmed the separation of the four different clades (Fig. 5b and c). According to the PLS-DA analysis, the first three components collectively explained 69.1% of the variance in this data model. The explained variance scree plot and loading plot for the PLS-DA analysis are shown in Supplementary Fig. S6. The November–January group separated from the other sample groups.

A qualitative distribution analysis of the 330 total detected mass features was conducted to investigate the mass feature distribution among the four defined sample groups (Supplementary Fig. S7). Visualization of the mass feature intersections revealed that 121 mass features were shared between all sample groups. Interestingly, the highest diversity was found in the February–March group, with 306 detected mass features accounting for 92.7% of all detected mass features. Notably, the February–March group also had most unique mass features. The November–January group had the lowest diversity with 162 mass features, suggesting that between the dormancy in November–January and the release of dormancy in February–March, the metabolite composition of the surface resin shifted drastically. Of the 330 total mass features, only 144 are shared between the November–January and February–March groups. Similarly, the February–March and the April and October groups shared 203 mass features, demonstrating a shift of 127 mass features. In pairwise comparison, the February–March and May–September groups demonstrated the most common mass features, consistent with the PLS-DA analysis for these overlapping groups.

**Figure 6.**
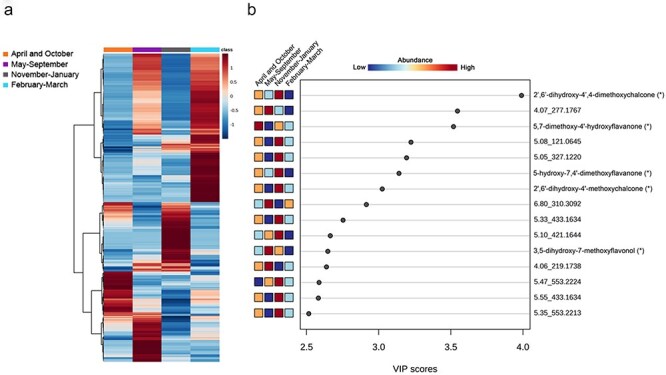
Metabolite distribution analysis of *P. trichocarpa* surface resin extracts. (a) Hierarchical cluster heatmap of mass feature distribution representing the relative abundance of 330 mass features between the different sample groups. Sample groups are indicated by color coding. (b) VIP scores for detected mass features. A higher VIP score signifies a higher importance on Component 1 of PLS-DA shown in (b). The colored boxes indicate the relative abundance of a mass feature between the sample groups. The mass features are annotated when available based on matching *m*/*z* of [M + H]^+^. Putatively identified mass features are indicated by *. Naming includes RT and *m*/*z* for unidentified mass features.

Similar to the whole leaf bud analysis, quantitative distribution analysis of mass features across the four sample groups revealed substantial shifts in metabolite profiles. Visualization of mass feature abundance based on sample groupings (Fig. 6a) displayed large, characteristic clades of mass features for each sample group. Based on the PLS-DA analysis, we identified mass features with the highest VIP scores on Component 1 (Fig. 6b), Component 2, and Component 3 (Supplementary Table S3). In surface resin, the top 15 mass features with the highest scores on Component 1 that were identified were *O*-methylated flavonoids belonging to the flavanone and chalcone groups. Among them, a mass feature 4.93_301.1063 had the highest VIP score. Two other mass features with similar mass but differing in retention time, 5.03_301.1064 and 5.40_301.1068, also showed high VIP scores. Together, the accurate masses of these mass features corresponded to a [M + H]^+^ of C_17_H_16_O_6_, consistent with 2ʹ,6ʹ-dihydroxy-4ʹ,4-dimethoxychalcone, 5-hydroxy-7,4ʹ-dimethoxyflavanone, and 5,7-dimethoxy-4ʹ-hydroxyflavanones. These identifications could not be confirmed due to the lack of analytical standards. However, 2ʹ,6ʹ-dihydroxy-4ʹ,4-dimethoxychalcone and 5-hydroxy-7,4ʹ-dimethoxyflavanone were previously reported to be produced by poplar in the literature (Greenaway et al. 1991a).

In addition, 2ʹ,6ʹ-dihydroxy-4ʹ-methoxychalcone was identified with high abundance in the November–January group, while a mass feature putatively annotated as 5,7-dimethoxy-4ʹ-hydroxyflavanone had the highest abundance in the April and October group. Furthermore, unidentified mass features 4.07_277.1767, 6.80_310.3092, and 4.06_219.1738, along with a putative 3,5-dihydroxy-7-methoxyflavonol, exhibited the highest VIP scores in the May–September group. The exact identity and the position of the *O*-methylation of the putative 3,5-dihydroxy-7-methoxyflavonol could not be verified as analytical standards were not available.

In total, 75 mass features were observed with a VIP score of ≥1 (Component 1), indicating their contribution to the differentiation between the surface resin sample groups (Supplementary Table S4). In addition to the described *O*-methylated flavonoids, salicinoids, including salicortin and tremulacin, and putative cinnamic acid esters, including cinnamyl caffeate, were identified. Dihydrochalcones scored high in VIP scores only when the mass features contributing to Component 2 were analyzed (Supplementary Tables S3B and S3C). These results indicate that other methoxylated flavonoids in addition to dihydrochalcones played a significant role in separating the sample groups that define the seasonal shifts in the bud surface resin.

## Discussion

Our seasonal metabolomic analysis of *P. trichocarpa* leaf buds and resin extends our knowledge of leaf bud resin chemistry on several fronts. First, we confirmed the quantitative importance of the methylated dihydrochalcones in *P. trichocarpa* leaf bud resin and established that the relative proportions of these dihydrochalcones in resin are constant throughout the seasons. Second, our seasonal time course suggests that dihydrochalcones and other resin components are actively synthesized from May to August during bud development, with very little or no synthesis during the fall, winter, and spring months. Metabolomic analysis of whole bud extracts revealed that sample groupings that confirm changes in bud chemistry occur during the summer months (June–August) and additional dynamics in chemistry composition in April–May during bud break. Third, the surface resin metabolomic analysis also indicated metabolite profile changes in secreted resin throughout the seasons, but with distinct patterns which suggest differential secretion or losses at the bud surface.

### *Populus trichocarpa* leaf bud extract shows a consistent seasonal dihydrochalcone profile

Dihydrochalcones are characteristic of *P. trichocarpa* resin but are absent in most other poplar species (English et al. 1991). The leaf bud dihydrochalcone profile in this study was dominated by methylated compounds, in particular, 2ʹ,4ʹ,6ʹ-OH-4-OMe dihydrochalcone, which comprised about half of all the dihydrochalcones present. As leaf buds grow and develop, the dihydrochalcone content increases dramatically, but the relative proportions of the identified dihydrochalcones remain constant, with 2ʹ,4ʹ,6ʹ-OH-4-OMe dihydrochalcone consistently being the most abundant. Nonmethylated dihydrochalcones make up a smaller proportion of these chemicals. Our method of quantification of dihydrochalcones is based on commercial standards and thus extends the earlier work of English et al. (1991) who reported relative abundance of dihydrochalcones based on GC–MS total ion current. Our determination of a dihydrochalcone content of up to 175 µg/mg resin dry weight underlines the quantitative importance of these compounds in *P. trichocarpa*. We recently identified the *O*-methyltransferase enzyme, using a gene expression profiling approach, which specifically methylates dihydrochalcones at both the 4 and 4ʹ positions and very likely generates the observed compounds (Piirtola et al., under revision). This enzyme does not act on other types of flavonoids including chalcones and thus appears to be specialized for *P. trichocarpa* resin.

Other types of flavonoids identified in this study are also methylated and lack glycosylation, consistent with the hydrophobic nature of the resin. Based on accurate masses and literature reports, we tentatively identified several methylated chalcones and flavanones (Figs. 4 and 6), which could follow similar seasonal dynamics as the dihydrochalcones, but this remains to be investigated. Similar *O*-methylated flavonoid aglycones from bud exudates have been previously described from other trees or shrubs, including alders (*Alnus* spp.), birches (*Betula* spp.), hop-hornbeams (*Ostrya* spp.), horse chestnuts (*Aesculus* spp.), buckthorns (*Rhamnus* spp.), starburs (*Acanthospermum* spp.), cherries (*Prunus* spp.), and *Decarya madagascariensis* (Wollenweber and Dietz 1981), confirming the importance of this type of compound for resin function.

### Resin and dihydrochalcone syntheses occur primarily during late summer in expanding lateral leaf buds

Our analysis of the leaf bud extracts indicates that dihydrochalcone biosynthesis in *P. trichocarpa* lateral leaf buds occurred primarily in the summer months of June–August, coinciding with leaf bud development. We note that for consistency, our analysis focused on the lateral (axillary) buds which form in early spring as the internodes expand. In these newly formed buds, dihydrochalcone content rose steadily on a tissue fresh weight basis until August (Fig. 2), suggesting active biosynthesis. Similarly, metabolomic analysis revealed distinct clusters representing the summer months but with June and July–August showing individual clusters. Expression of the *P. trichocarpa* DHC *O*-methyltransferase mentioned earlier is consistent with this pattern: its transcript levels were minimal throughout the year, rise steeply in June, peak in July and August, and then fall off rapidly in September (Piirtola et al., under revision). This biosynthetic phase of bud chemistry reflects the development of embryonic leaves and bud scales, structures which contribute to resin synthesis. In the closely related poplar *P. deltoides*, the base of the leaf bud scales develops a specialized epidermal cell layer on the adaxial surface, suggesting a secretory function (Curtis and Lersten 1974). Our observations for *P. trichocarpa* buds suggest a similar structure for the bud scales in this species. The distinct June sample cluster observed in the metabolomics analysis suggests that this month is associated with a unique leaf bud resin composition that was not captured in the targeted dihydrochalcone analysis (Fig. 3). June is a period of leaf bud maturation, and one would expect there to be many new primary or secondary metabolites at this time. Such metabolites could include not only additional secondary metabolites (see below) but also sugars, amino acids, and small lipids. Their identification was beyond the scope of this work, however.

Following the period of rapid synthesis in the summer months, the leaf bud metabolome became static from September onward through the fall and winter months as evidenced by the consistent quantity of dihydrochalcones (Fig. 2) and the grouping of samples from September to March in the metabolomic analyses (Fig. 3). The September through March timeframe corresponds to bud dormancy during fall and winter months when metabolic activity in buds is expected to be low. The grouping of April and May samples from the PLS-DA is surprising and not readily explained, since these samples represented the newly formed buds in May, with the expanding previous year’s flushed buds in April. However, both time points show the lowest abundance scores for compounds contributing to a high VIP ranking (Fig. 4). The low abundance would contribute heavily to sample grouping in the analysis. The May samples comprised very young buds (∼2 mm in length), which may not have had sufficient time to synthesize much resin. In contrast, decline in total dihydrochalcone concentration and resin in April could be due to the increasing proportion of leaf tissue in the samples during bud break when leaves expand and accumulate water. The increase in tissue mass would dilute the concentration of dihydrochalcones and other resin metabolites. Such a dilution effect was previously observed in *Betula* spp, where flavonoid content decreased with the increasing leaf size and a reduction in the density of the secretory trichomes on the leaf surface (Valkama et al. 2004).

The observed seasonal dynamics of bud resin chemistry are generally consistent and reflect rapid synthesis in the summer, stable metabolite composition through the winter, and dynamic changes in the spring. Nevertheless, as outlined earlier, we observed some differences in patterns of dihydrochalcone accumulation and the sample groupings derived from the PLS-DA and hierarchical clusters. Which molecules might drive the clustering and explain such differences can be gleaned from the VIP analysis, which ranks specific molecules or features according to their impact on the groupings: while dihydrochalcones rank highly in the VIP analysis, other molecules also appear within the top-ranked features (Fig. 4). As mentioned earlier, some of these were identified as methylated flavonols and chalcones; eight of the top 15 ranked compounds are derived from the flavonoid pathway, and all but one are methylated. This underlines the importance of methylation and the flavonoid pathway for the observed dynamics of *P. trichocarpa* resin.

### Changes in bud surface resin are not tightly coupled to resin synthesis and could reflect constraints on secretion and the loss of volatiles

The patterns observed for the leaf bud surface resin provided a contrast to that of whole leaf bud extracts. The dihydrochalcone analysis showed more quantitative variation, and the nontargeted metabolomic analysis gave rise to different clusters and groupings. We note that due to varying bud sizes, surface resin samples were normalized by extract weight rather than tissue fresh weight as for the whole bud extracts. This makes direct comparison of the bud extracts with surface resin more difficult, although patterns in the seasonal profiles are still informative. A second caveat is that the surface washes to collect the bud surface resin for the May–July time points included expanded leaf tissue in addition to the newly formed buds, and this may have influenced some of our results. However, when we repeated the hierarchical clustering and PLS-DA analyses without these sample points, similar sample cluster groupings emerged (Supplementary Fig. S8), although the spring and summer samples separated along a third component. Overall, these differences did not alter interpretation of the data.

A comparison of the nontargeted and targeted data sets shows clear parallels in seasonal dynamics. For example, as in whole bud extracts, the surface resin dihydrochalcone content was low during the summer months. However, it remained low for a longer time, extending into August and September. This likely reflects the temporal delay between synthesis and dihydrochalcone movement or secretion to the bud surface (see below). The dihydrochalcone content of surface resin did show an upward though nonsignificant trend from January to March, followed by a sharper increase in April. This was in contrast to the strong downward trend of dihydrochalcone concentration in whole bud extracts at this time point (Fig. 2). We speculate that in April, the opening of the bud allows the extraction solvent greater access to the expanding leaves and scales inside the bud, more effectively extracting dihydrochalcones and other compounds in surface resin. However, we cannot rule out some synthesis of dihydrochalcone as the buds expand. Our previous work on dihydrochalcone *O*-methyltransferase expression suggests that there is a low level of dihydrochalcone synthesis in April during bud burst, though at much lower levels than in the summer months (Piirtola et al., submitted).

A second spike in dihydrochalcone content of surface resin occurred in October. Interestingly, the hierarchical cluster analysis grouped this time point with April, presumably due to the high dihydrochalcone content (∼17% of resin dry weight) and other resin components in those months. The reason for the increase in October is not clear, but it could involve the differential loss or volatilization of other resin components that lead to dihydrochalcone enrichment. *Populus trichocarpa* resin in particular contains large quantities of volatile benzenoids such as benzyl benzoate, benzyl salicylate, and benzyl 2-methylbenzoate, as well as terpenoids (English et al. 1991, Pobłocka-Olech et al. 2024). Weather records suggest a warm period during the October sampling period, which could enhance this effect. This is also the time of abscission and leaf drop, and physiological changes at this time may lead to an increase in dihydrochalcone and resin secretion prior to the onset of dormancy.

Overall, inspection of the metabolite patterns of whole bud extract and surface resin data sets indicates that the secretion of resin is shifted relative to its biosynthesis. Mechanistically, this makes sense, since resin synthesis occurs within buds by the concentrically arranged bud scales and by very young leaves which develop inside the bud (Curtis and Lersten 1974). Resin has to move from the inside of the cell and pass through the cell walls and cuticle before it is released onto the inner surface of the bud scale. From there, resin would have to flow to the outside of the bud. How resin secretion from cells in the scales occurs has not been studied, but mechanical forces from cellular expansion and contraction are proposed as a potential mechanism (Paiva 2016).

Our nontargeted metabolomic analyses of the surface resin only partially reflected the dynamics of dihydrochalcone content, suggesting that other phytochemicals besides the dihydrochalcones are driving the sample clustering. Our VIP analysis supports this idea, and the most important compounds for explaining surface resin dynamics are methylated chalcones, flavanones, and flavanols, rather than dihydrochalcones (Fig. 6, Supplementary Table S4). In future studies, quantification of these flavonoids to assess differences in their accumulation patterns should be investigated. Tracking the seasonal shifts in these additional compounds in bud resin will be important for a more detailed understanding of the drivers of resin dynamics.

## Conclusions

This study describes the first investigation on the dynamics of metabolite accumulation in *P. trichocarpa* leaf bud resin. The accumulation of dihydrochalcones is a dominant factor driving seasonal dynamics in whole buds, although other methylated flavonoids are also important. The dynamics of surface resin are shifted relative to whole bud extracts and are less impacted by dihydrochalcone accumulation. While our work focused on nonvolatile components of resin, it provides a first look at seasonal changes. The imperfect alignment of the phytochemical patterns observed in whole leaf buds versus the surface resin emphasizes the need to investigate the mechanisms of leaf bud resin diffusion and secretion in more detail.

## Materials and Methods

### Collection and extraction of *P. trichocarpa* leaf bud resin

Poplar leaf bud samples of *P. trichocarpa* (Nisqually-1) were collected from the University of Victoria Research Compound (48°27ʹ N, −123°18ʹ W). Intact leaf buds of *P. trichocarpa* were collected every month (mid-month) for 12 months from three adjacent clonal trees (biological replicates) (Supplementary Fig. S1). For leaf bud extraction, 10 lateral leaf buds were sampled from each biological replicate tree and frozen immediately in liquid nitrogen. For the collection of surface resin, three replicate sets of five similarly sized lateral leaf buds were collected from individual branches of each tree. Surface resin between May and July was collected from the entire leaf nodes, which included new buds as well as resinous expanding leaves, due to the very small size of the developing buds.

The surface resin extracts of *P. trichocarpa* leaf buds were collected by accurately weighing 0.5 g (fresh weight) of intact lateral leaf buds and submerging them in 0.1 ml of HPLC-grade methanol per 10 mg of sample. The leaf buds were stirred for 1 min, after which the supernatant was collected. The methanol extract was filtered using a 0.20-µm polytetrafluoroethylene filter to remove any solid particles. The extract was dried by transferring 2 ml of methanol extract into preweighed Eppendorf tubes and evaporating the samples using an Eppendorf concentrator until dry. The dried samples were accurately weighed to determine the dry weight of the surface resin extracts. The dry extracts were stored at −20°C prior to the analysis.

For whole bud extracts, harvested leaf buds were homogenized into a fine powder in liquid nitrogen using a ceramic mortar and pestle and stored at −80°C before extraction. The powder was accurately weighed to 40 mg (fresh weight), and 1 ml of methanol was added. Samples were vortexed, sonicated for 10 min in a sonicating water bath, and centrifuged for 10 min at 15 000 rpm. The supernatant was collected, and the extraction was repeated on the remaining plant tissue pellet. Supernatants from both extractions were combined, and the pooled samples were dried using a SpeedVac for 2 h until dry. The dried extracts were stored at −20°C prior to analysis. For nontargeted analysis, nine extraction blanks without tissue were prepared using the same protocol.

### Targeted quantification of dihydrochalcones in *P. trichocarpa* in leaf bud resin extracts by UPLC–MS

Whole leaf bud extracts were reconstituted in 1 ml of methanol. These samples were normalized by the fresh weight of the extracted whole buds. Surface resin samples were normalized by the extract dry weight and reconstituted to 100 µg/ml. The samples were analyzed using a Waters Acquity UPLC System coupled to an Acquity PDA eLambda Detector and an Acquity QDa single quadrupole mass spectrometer (Waters, Milford, MA, USA). The column used for separation was Acquity BEH C_18_ (50 mm × 2.1 mm ID, 1.7 µm). The column manager temperature was set to 40°C, and the autosampler temperature was 10°C. The two-solvent gradient consisted of Solvent A [ddH_2_O with 0.1% formic acid (v/v)] and Solvent B [acetonitrile with 0.1% formic acid (v/v)] at a flow rate of 0.5 ml/min. The gradient is as follows: 0.1% B (0–0.5 min), 0.1–50% B (0.5–5 min), 50–90% B (5–8 min), 90% B column wash (8–9 min), 90–0.1% B (9–9.1 min), and 0.1% B for column equilibration (9.1–11 min). The UV detector range was 190–800 nm. The MS conditions are as follows: capillary voltage 0.8 kV, probe temperature 600°C, source temperature 150°C, and cone gas (N_2_) flow. The MS full scan range was 50–1000 *m*/*z*, and the MS analysis was performed using the negative ionization mode.

Analytical standards for 2ʹ,4ʹ,6ʹ-trihydroxydihydrochalcone (2ʹ,4ʹ,6ʹ-OH DHC), 2ʹ,4ʹ,6ʹ-trihydroxy-4-methoxydihydrochalcone (2ʹ,4ʹ,6ʹ-OH-4-OMe DHC), 2ʹ,6ʹ-dihydroxy-4,4ʹ-dimethoxydihydrochalcone (2ʹ,6ʹ-OH-4,4ʹ-OMe DHC), salicortin, and tremulacin were purchased from Biosynth Carbosynth (San Diego, USA). Phloretin (2ʹ,4ʹ,6ʹ,4-OH DHC), asebogenin (2ʹ,6ʹ,4-OH-4ʹ-OMe DHC), and 2ʹ,6ʹ-dihydroxy-4ʹ-methoxydihydrochalcone (2ʹ,6ʹ-OH-4ʹ-OMe DHC) were purchased from TransMIT (Giessen, Germany). Pinocembrin chalcone and naringenin chalcone were purchased from Extrasynthese (Lyon, France). Salicin was purchased from Sigma-Aldrich (Oakville, Canada).

To collect compound-specific MS data, selected ion recording methods were optimized for the predominant molecular ion [M − H]^−^ of each compound of interest. The optimized parameters are as follows: phloretin (273 *m*/*z*, 15 V), asebogenin (287 *m*/*z*, 15 V), 2ʹ,4ʹ,6ʹ-trihydroxydihydrochalcone (257 *m*/*z*, 15 V), 2ʹ,6ʹ-dihydroxy-4ʹ-methoxydihydrochalcone (271 *m*/*z*, 15 V), 2ʹ,6ʹ-dihydroxy-4,4ʹ-dimethoxydihydrochalcone (301 *m*/*z*, 15 V), and 2ʹ,4ʹ,6ʹ-trihydroxy-4-methoxydihydrochalcone (285 *m*/*z*, 15 V). Calibration curves for each compound were prepared with a concentration range of 0.05–100 µg/ml. MS data were processed, and the peak areas were integrated using TargetLynx (Version 4.2).

### Nontargeted metabolomics of *P. trichocarpa* leaf bud resin extracts

UPLC–HRMS analyses of poplar leaf bud extracts were carried out as previously described by Witte, et al. (2021). Samples were resuspended in methanol based on extract dry weight (surface resin samples) or fresh tissue weight (whole leaf bud extract) to yield a final concentration of 500 µg/ml. Nine extraction blanks and seven methanol blanks were used as quality controls for data preprocessing, and samples were analyzed in a randomized injection order. Chromatographic separation of the samples was achieved on a Thermo Ultimate 3000 UPLC coupled to a Thermo LTQ Orbitrap XL high-resolution mass spectrometer. The UPLC–HRMS analysis was carried out using a Phenomenex C_18_ Kinetex column (50 mm × 2.1 mm ID, 1.7 µm). The two-solvent gradient for the flow gradient consisted of Solvent A [ddH_2_O with 0.1% formic acid (v/v)] and Solvent B [acetonitrile with 0.1% formic acid (v/v)] at a flow rate of 0.35 ml/min. The gradient is as follows: 1% B (0–0.5 min), 1–99% B (0.5–4.5 min), 99% B (4.5–8 min), 99–1% B (8–8.5 min), and 1% B (8.5–11.5 min) to equilibrate the column to starting conditions. The electron spray ion source for the MS analysis used the following parameters: a capillary voltage of 34 kV, an ionization voltage of 4.0 kV, a tube lens voltage of 100 V, sheath gas 40, auxiliary gas 5, and sweep gas 2. The MS full scan range was 100–2000 *m*/*z*, and the MS analysis was performed using positive ionization modes. Thermo Fisher Xcalibur (version 4.6.6717) was used for reviewing the raw data files.

#### Metabolomics data processing.

The raw UPLC–HRMS data files were processed using MZmine2 (version 2.53) (Pluskal et al. 2010). Data preprocessing algorithms included mass detection, an Automated Data Analysis Pipeline (ADAP) chromatogram builder, a local minimum search for chromatogram deconvolution, an isotopic peak grouper, peak alignment, and gap filling. Data preprocessing parameters are described in detail in Supplementary Table S5. In summary, UPLC–HRMS raw data files were used to determine the noise level cutoff for the full width at half maximum exact mass algorithm for mass detection. The ADAP chromatogram builder was used to build a chromatogram for each mass feature detected continuously over the scans. The group intensity threshold was set to noise level, and the minimum highest intensity was set to three times the noise level, with mass tolerance set to 5 ppm. Chromatogram deconvolution was performed using a local minimum search algorithm and by optimizing the algorithm parameters by previewing the data files. The Isotopic Peak Grouper algorithm was used to find peaks that form an isotope pattern and to remove isotopes besides the highest one. The join aligner method was used for signal alignment with a mass tolerance of 5 ppm, weight for *m*/*z* to RT ratio of 20:10, and RT tolerance of 0.1 min. The peak finder method was used for gap filling to review the aligned data and fill in the detected mass variable values below the noise threshold.

Preprocessed sample data were curated in Microsoft Excel and visualized by plotting a heatmap highlighting mass features detected above the noise level threshold. As part of data curation, duplicate mass features were removed. The mass features above the noise level in extraction and methanol blanks were eliminated from the data set to account for contaminations.

#### Mass feature annotation.

Detected mass features were defined by RT and exact mass (RT_*m*/*z*). Mass features corresponding to dihydrochalcones were identified by comparison of the exact *m*/*z* and RT to analytical standards. Unknown mass features were annotated by comparing their observed exact *m*/*z* to the theoretical *m*/*z* of compounds previously reported in the poplar literature (Greenaway et al. 1989, 1992, English et al. 1991, 1992, Nissinen et al. 2017, Gordon et al. 2022). Pseudo-molecular ionic annotations were assigned to mass features with an identical mass (mass error < 5 ppm) compared to predicted structures. The complete total ion chromatogram data matrixes are shown in **Supplementary Tables S6 and S7**.

### Statistical analysis

For the statistical analysis of the nontargeted metabolomic samples, the data were normalized by sample mass and centered using Pareto scaling. Hierarchical cluster analysis, principal component analysis, and univariate and multivariate analyses were conducted using MetaboAnalyst 5.0 (Pang, et al., 2021). Statistical analyses, including two-way ANOVA and Tukey’s honestly significant difference (HSD), were performed using GraphPad Prism (Version 8.4.3). Visualization of the intersecting mass features between the sample groups from the qualitative differential analysis was performed using the UpSet package on R (Lex et al. 2014).

## Supplementary Material

pcae149_Supp

## Data Availability

The data are available through the links provided in the manuscript. Raw nontargeted metabolomics data are available at Metabolomics Workbench (https://www.metabolomicsworkbench.org/, Study ID ST003560, doi: 10.21228/M81C1C).
