# Supplementary material for: Poplar leaf bud resin metabolomics: seasonal profiling of leaf bud chemistry in Populus trichocarpa provides insight into resin biosynthesis
Source: Plant Cell Physiol. 2024 Dec 19;66(3):291–303. doi: 10.1093/pcp/pcae149 (PMC11957272; doi:10.1093/pcp/pcae149)
Supplement: pcae149_Supp [file pcae149_supp.zip › suppl_data/pcp-2024-e-00152-File003.pptx]

## Slide 1
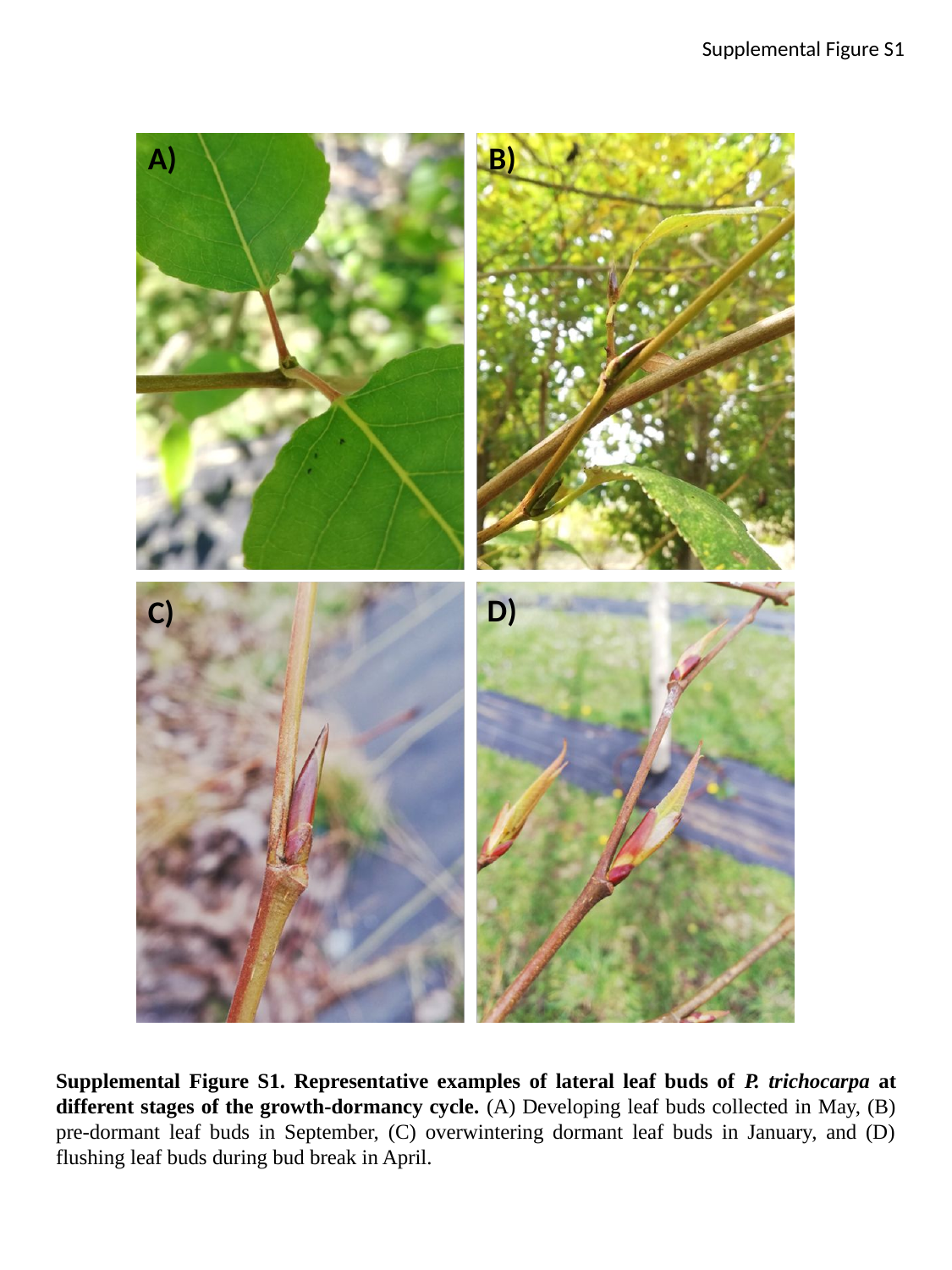

Supplemental Figure S1
A)
B)
D)
C)
Supplemental Figure S1. Representative examples of lateral leaf buds of P. trichocarpa at different stages of the growth-dormancy cycle. (A) Developing leaf buds collected in May, (B) pre-dormant leaf buds in September, (C) overwintering dormant leaf buds in January, and (D) flushing leaf buds during bud break in April.

## Slide 2
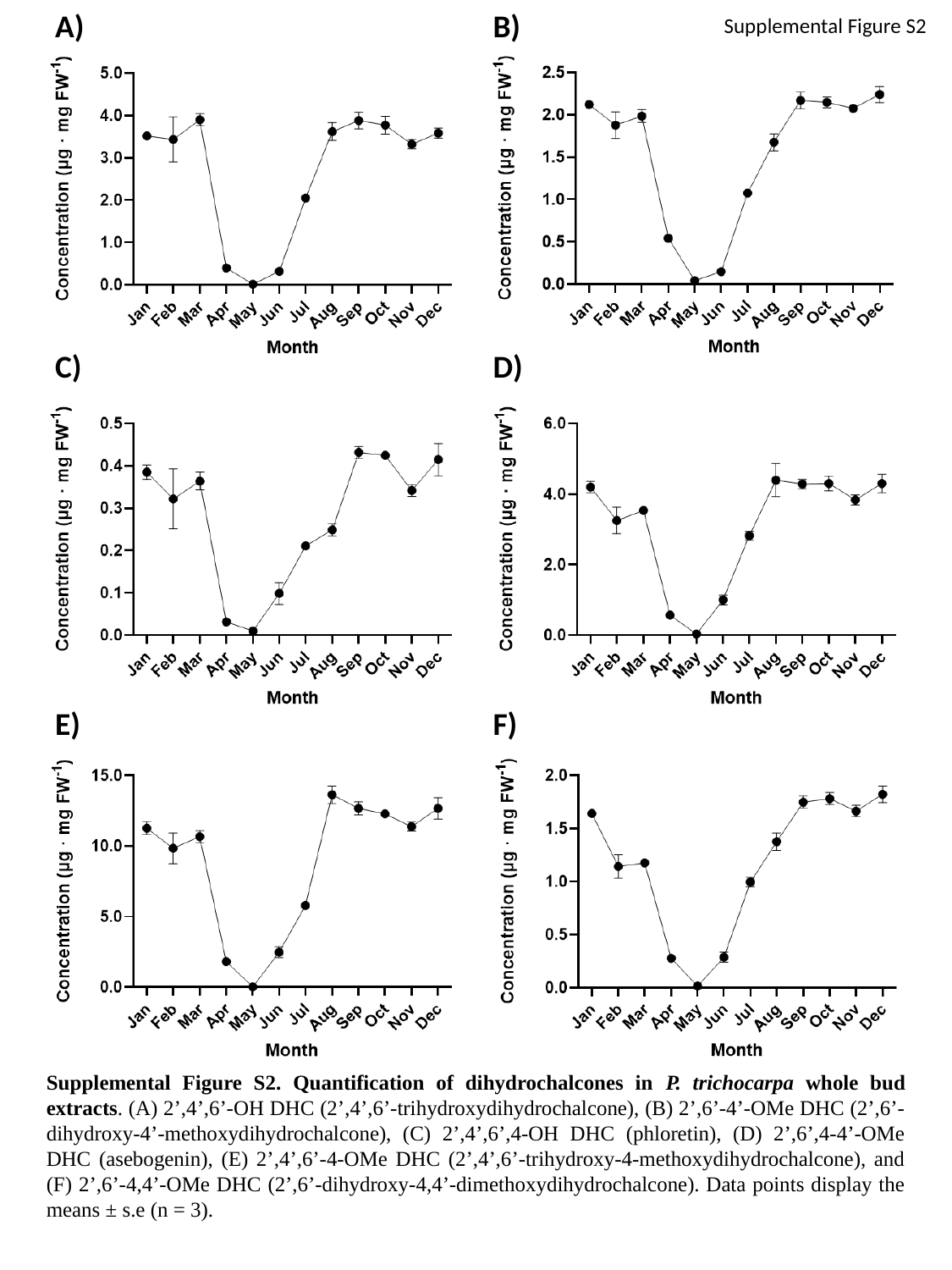

A)
B)
C)
D)
E)
F)
Supplemental Figure S2
Supplemental Figure S2. Quantification of dihydrochalcones in P. trichocarpa whole bud extracts. (A) 2’,4’,6’-OH DHC (2’,4’,6’-trihydroxydihydrochalcone), (B) 2’,6’-4’-OMe DHC (2’,6’-dihydroxy-4’-methoxydihydrochalcone), (C) 2’,4’,6’,4-OH DHC (phloretin), (D) 2’,6’,4-4’-OMe DHC (asebogenin), (E) 2’,4’,6’-4-OMe DHC (2’,4’,6’-trihydroxy-4-methoxydihydrochalcone), and (F) 2’,6’-4,4’-OMe DHC (2’,6’-dihydroxy-4,4’-dimethoxydihydrochalcone). Data points display the means ± s.e (n = 3).

## Slide 3
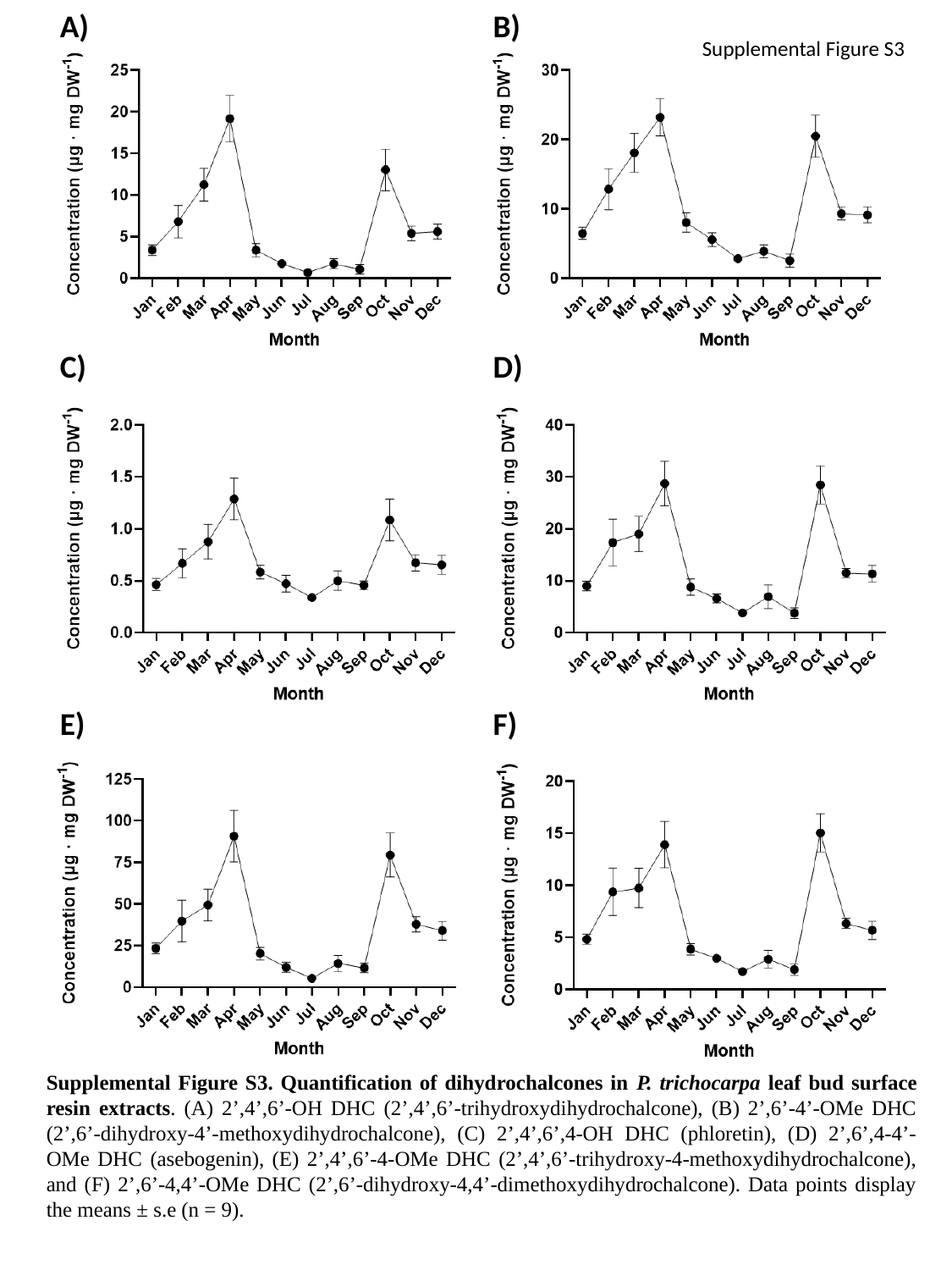

A)
B)
C)
D)
E)
F)
Supplemental Figure S3
Supplemental Figure S3. Quantification of dihydrochalcones in P. trichocarpa leaf bud surface resin extracts. (A) 2’,4’,6’-OH DHC (2’,4’,6’-trihydroxydihydrochalcone), (B) 2’,6’-4’-OMe DHC (2’,6’-dihydroxy-4’-methoxydihydrochalcone), (C) 2’,4’,6’,4-OH DHC (phloretin), (D) 2’,6’,4-4’-OMe DHC (asebogenin), (E) 2’,4’,6’-4-OMe DHC (2’,4’,6’-trihydroxy-4-methoxydihydrochalcone), and (F) 2’,6’-4,4’-OMe DHC (2’,6’-dihydroxy-4,4’-dimethoxydihydrochalcone). Data points display the means ± s.e (n = 9).

## Slide 4
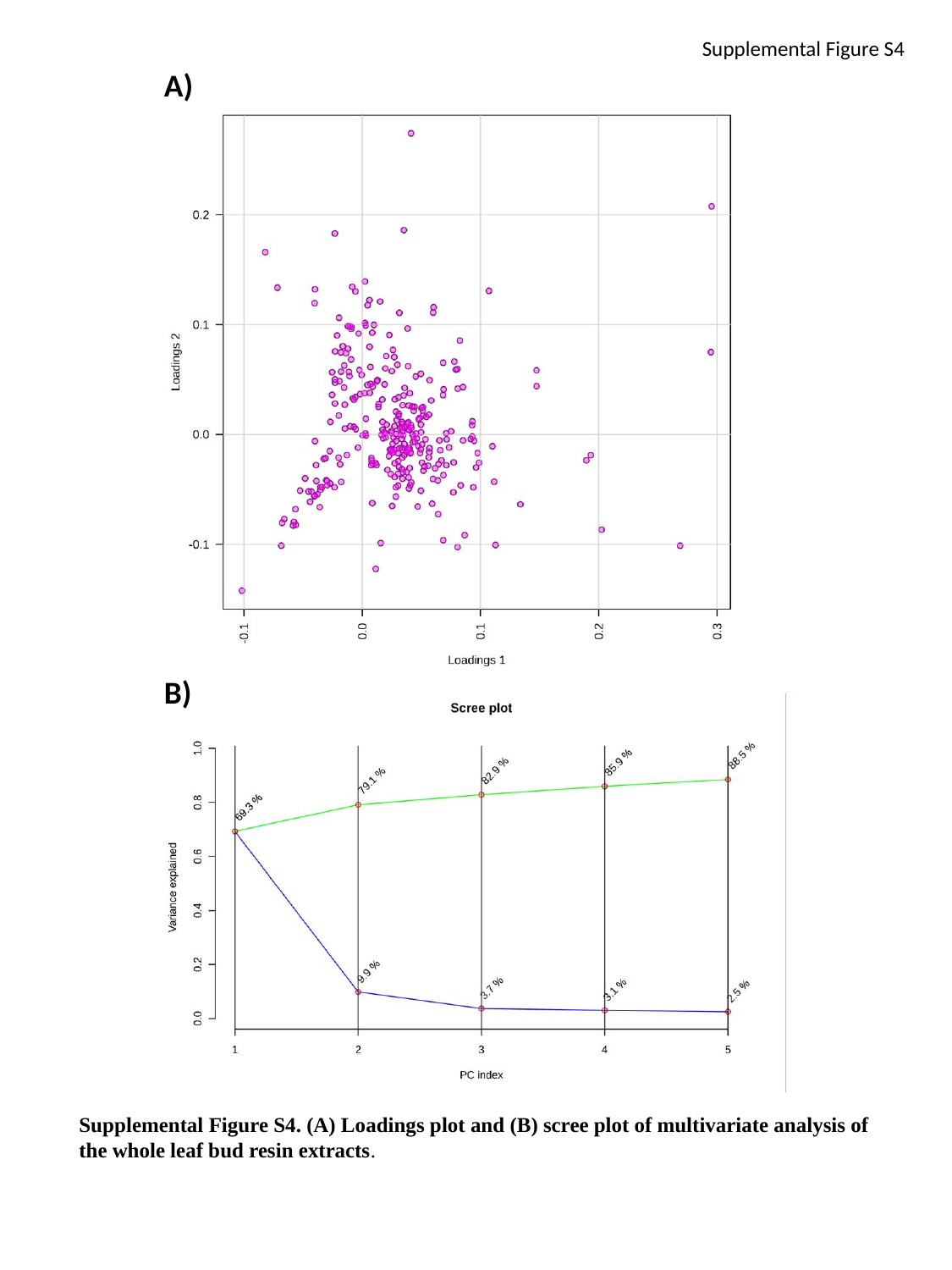

Supplemental Figure S4
A)
B)
Supplemental Figure S4. (A) Loadings plot and (B) scree plot of multivariate analysis of the whole leaf bud resin extracts.

## Slide 5
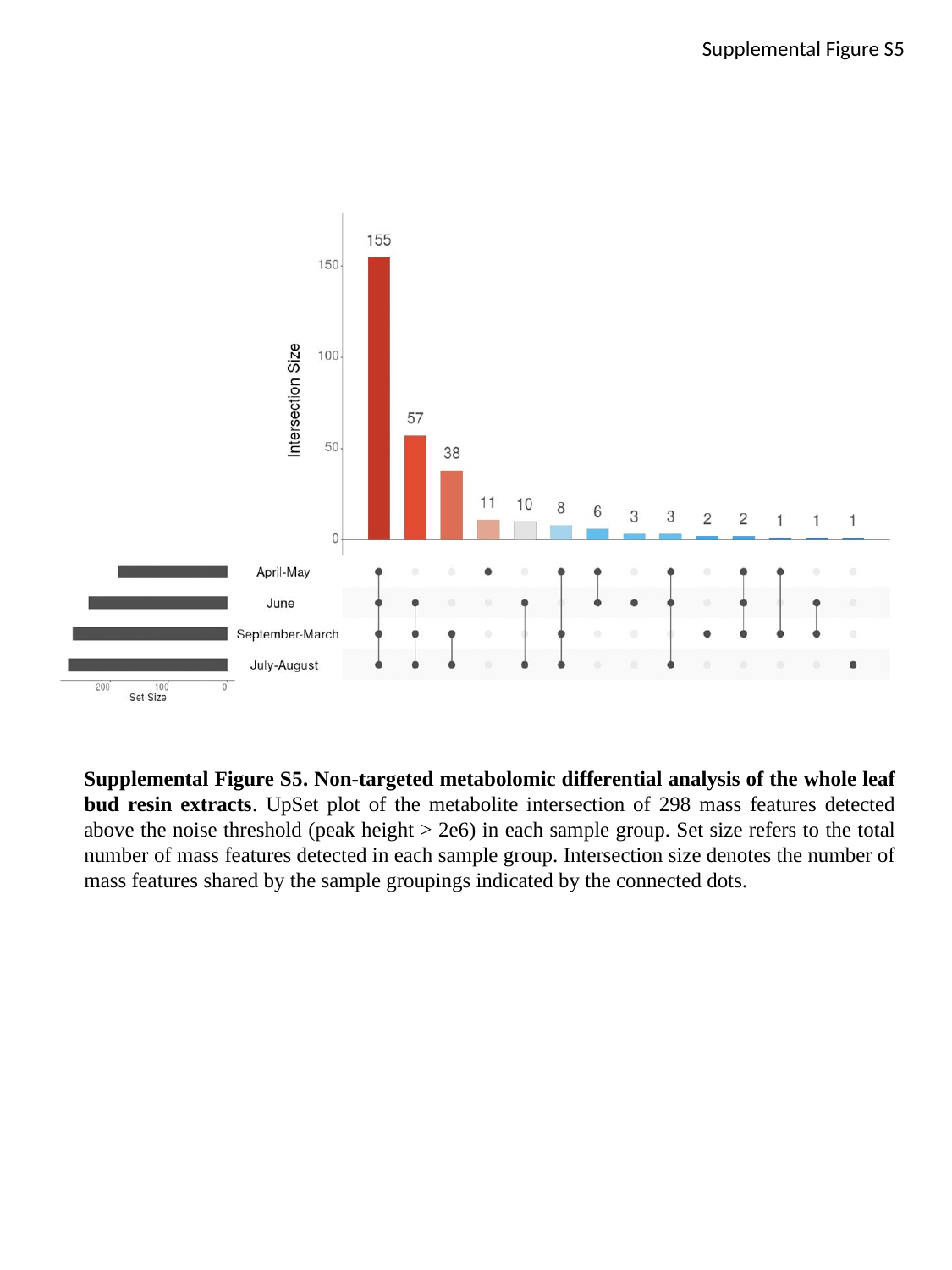

Supplemental Figure S5
Supplemental Figure S5. Non-targeted metabolomic differential analysis of the whole leaf bud resin extracts. UpSet plot of the metabolite intersection of 298 mass features detected above the noise threshold (peak height > 2e6) in each sample group. Set size refers to the total number of mass features detected in each sample group. Intersection size denotes the number of mass features shared by the sample groupings indicated by the connected dots.

## Slide 6
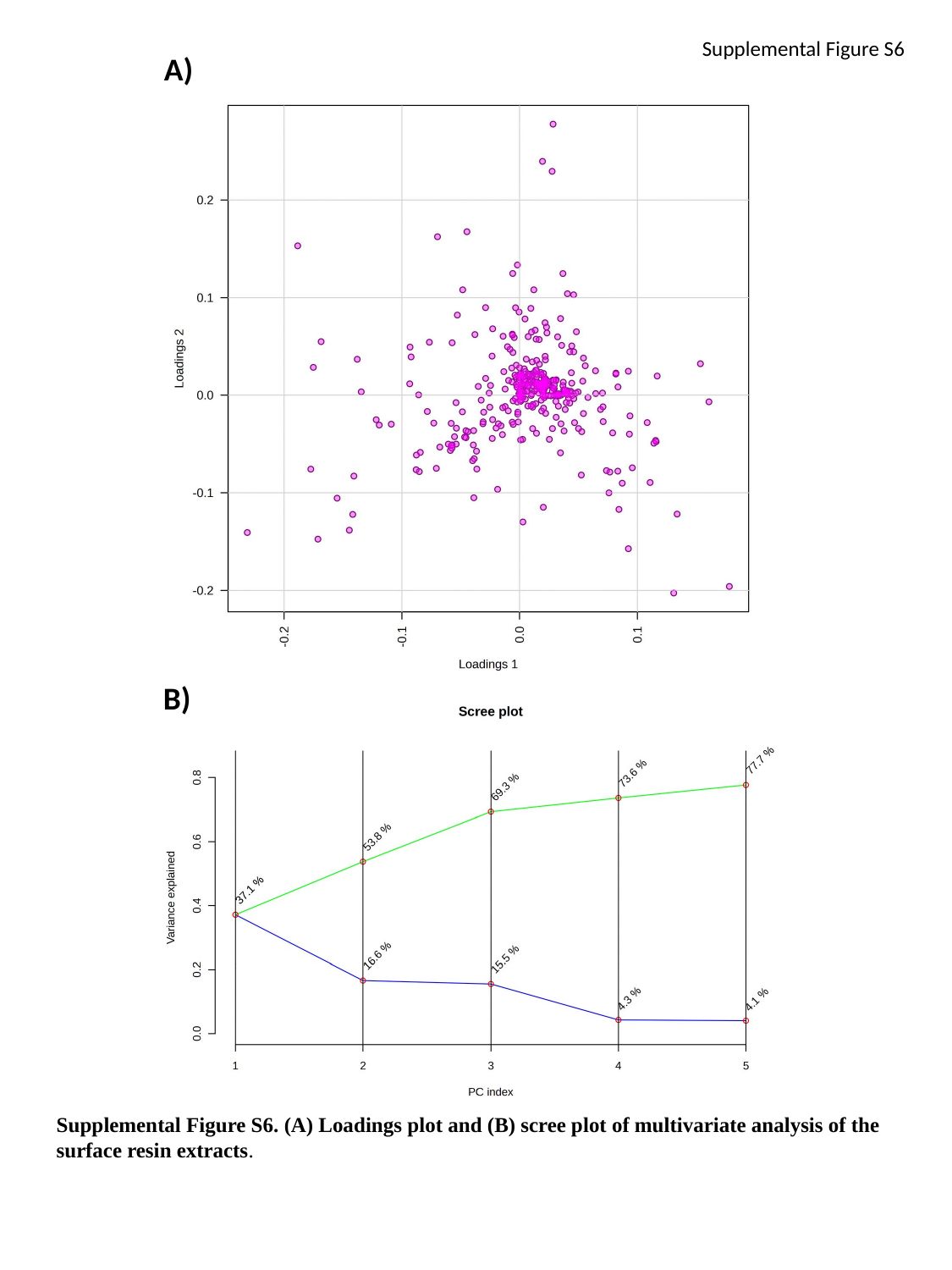

Supplemental Figure S6
A)
B)
Supplemental Figure S6. (A) Loadings plot and (B) scree plot of multivariate analysis of the surface resin extracts.

## Slide 7
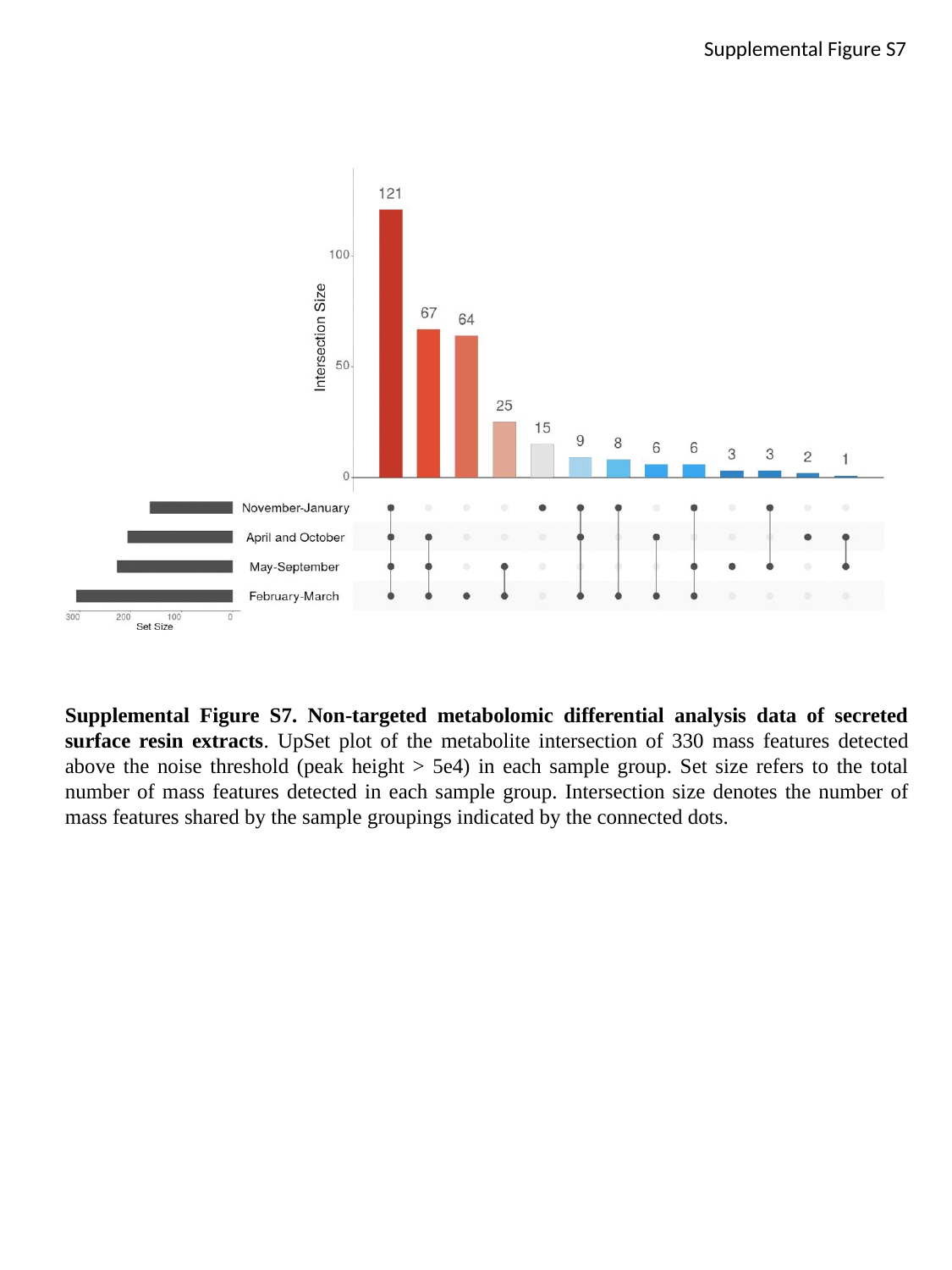

Supplemental Figure S7
Supplemental Figure S7. Non-targeted metabolomic differential analysis data of secreted surface resin extracts. UpSet plot of the metabolite intersection of 330 mass features detected above the noise threshold (peak height > 5e4) in each sample group. Set size refers to the total number of mass features detected in each sample group. Intersection size denotes the number of mass features shared by the sample groupings indicated by the connected dots.

## Slide 8
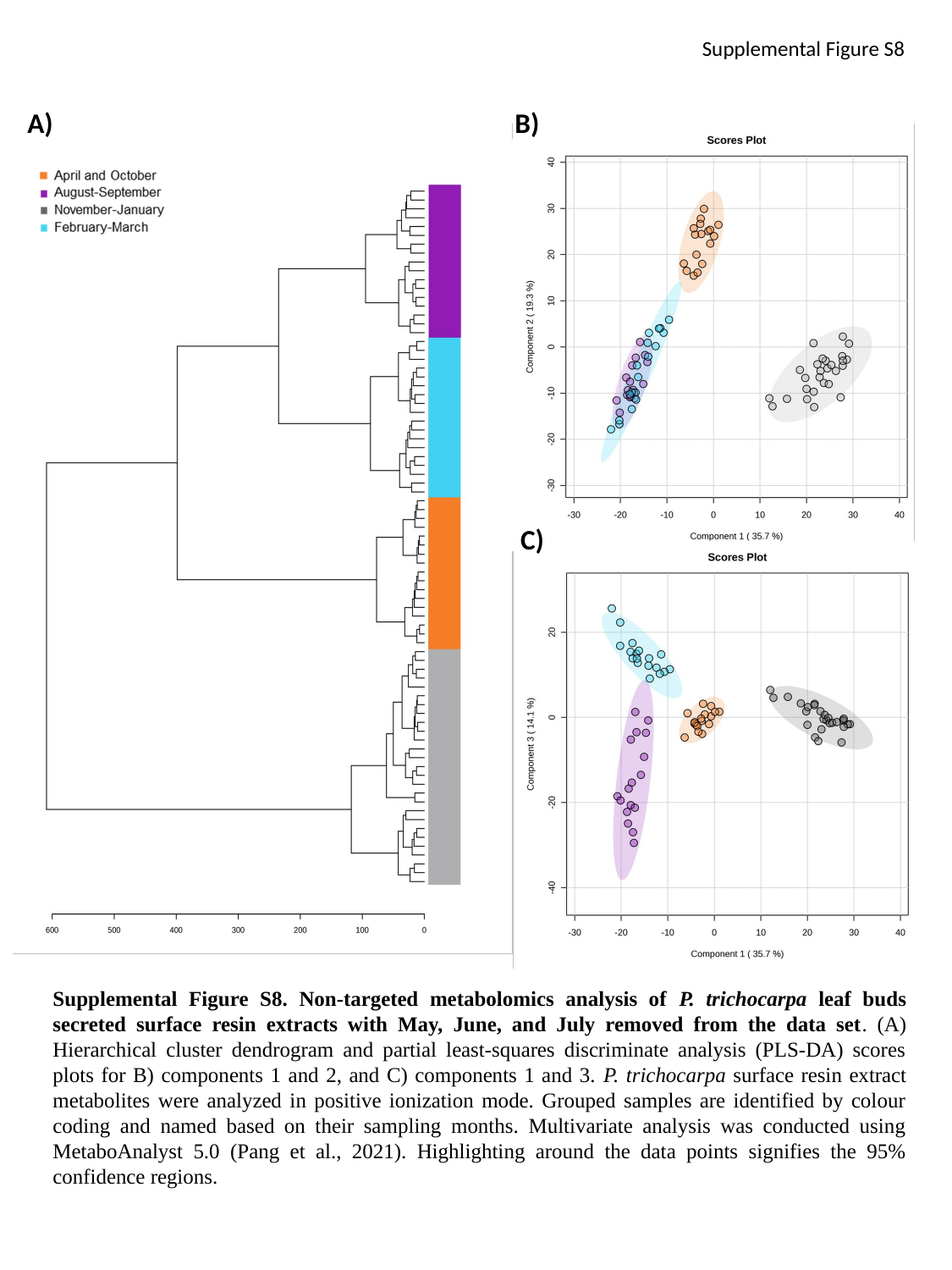

Supplemental Figure S8
A)
B)
C)
Supplemental Figure S8. Non-targeted metabolomics analysis of P. trichocarpa leaf buds secreted surface resin extracts with May, June, and July removed from the data set. (A) Hierarchical cluster dendrogram and partial least-squares discriminate analysis (PLS-DA) scores plots for B) components 1 and 2, and C) components 1 and 3. P. trichocarpa surface resin extract metabolites were analyzed in positive ionization mode. Grouped samples are identified by colour coding and named based on their sampling months. Multivariate analysis was conducted using MetaboAnalyst 5.0 (Pang et al., 2021). Highlighting around the data points signifies the 95% confidence regions.
