# Supplementary material for: Poplar leaf bud resin metabolomics: seasonal profiling of leaf bud chemistry in Populus trichocarpa provides insight into resin biosynthesis
Source: Plant Cell Physiol. 2024 Dec 19;66(3):291–303. doi: 10.1093/pcp/pcae149 (PMC11957272; doi:10.1093/pcp/pcae149)
Supplement: pcae149_Supp [file pcae149_supp.zip › suppl_data/pcp-2024-e-00152-File004.docx]

**Supplemental Table S1. A shortlist of the variable importance in projection (VIP) scores and loading values for mass features detected in whole leaf buds**. Pseudomolecular ion annotations for the 15 mass features with the highest VIP scores based on PLS-DA analysis of whole leaf buds presented in Fig. 3B for (A) component 1 and (B) component 2. When available, the mass features are annotated based on matching *m/z* and retention time (RT) of analytical standards, including 2’,4’,6’-OH-4-OMe DHC (2’,4’,6’-trihydroxy-4-methoxydihydrochalcone), 2’,6’-OH-4’-OMe DHC (2’,6’-dihydroxy-4’-methoxydihydrochalcone), 2’,4’,6’-OH DHC (2’,4’,6’-trihydroxydihydrochalcone), 2’,6’,4-OH-4’-OMe DHC (asebogenin), and 2’,6’-OH-4,4’-Me DHC (2’,6’-dihydroxy-4,4’-dimethoxydihydrochalcone). Putatively identified mass features based on accurate mass are indicated by (*). Component 1 explained 69.2 %, and component 2 explained 9.8 % of the variance in the PLS-DA data model.

| **RT** | ***m/z*** | **Pseudomolecular Ion Annotation** | **VIP score** | **Loadings 1** | **Loadings 2** |
| --- | --- | --- | --- | --- | --- |
| 4.74 | 289.1070 | 2’,4’,6’-OH-4-OMe DHC [M+H]^+^ | 4.99 | 0.29 | 0.07 |
| 4.80 | 285.0762 | 3,5-dihydroxy-7-methoxyflavonol [M+H]^+^ (*) | 4.66 | 0.27 | -0.10 |
| 4.72 | 287.0915 | 2’,4’,6'-trihydroxy-4-methoxychalcone [M+H]^+^ (*) | 4.60 | 0.30 | 0.21 |
| 5.20 | 273.1123 | 2’,6’-OH-4’-OMe DHC [M+H]^+^ | 3.55 | 0.20 | -0.09 |
| 4.79 | 259.0967 | 2’,4’,6’-OH DHC [M+H]^+^ | 3.23 | 0.19 | -0.02 |
| 5.16 | 271.0968 | 2',6'-dihydroxy-4'-methoxychalcone [M+H]^+^ (*) | 3.22 | 0.19 | -0.02 |
| 4.67 | 289.1069 | 2’,6’,4-OH-4’-OMe DHC [M+H]^+^ | 2.51 | 0.15 | 0.04 |
| 4.74 | 121.0649 | Unknown | 2.48 | 0.15 | 0.06 |
| 5.52 | 285.1125 | Unknown | 2.39 | 0.13 | -0.06 |
| 3.60 | 441.1758 | Unknown | 2.08 | 0.11 | -0.04 |
| 4.64 | 221.1175 | Unknown | 2.03 | 0.11 | -0.10 |
| 5.14 | 303.1227 | 2’,6’-OH-4,4’-Me DHC [M+H]^+^ | 1.90 | 0.11 | -0.01 |
| 3.61 | 441.1750 | Unknown | 1.86 | 0.09 | -0.09 |
| 4.55 | 205.1952 | Unknown | 1.74 | 0.10 | -0.03 |
| 4.24 | 267.0864 | Unknown | 1.74 | -0.10 | -0.14 |
|  | | |  |  |  |

**A)**

**B)**

| **RT** | ***m/z*** | **Pseudomolecular Ion Annotation** | **VIP score** | **Loadings 1** | **Loadings 2** |
| --- | --- | --- | --- | --- | --- |
| 4.74 | 289.1070 | 2’,4’,6’-OH-4-OMe DHC [M+H]^+^ | 4.95 | 0.29 | 0.07 |
| 4.80 | 285.0762 | 3,5-dihydroxy-7-methoxyflavonol [M+H]^+^ (*) | 4.63 | 0.27 | -0.10 |
| 4.72 | 287.0915 | 2’,4’,6'-trihydroxy-4-methoxychalcone [M+H]^+^ (*) | 4.59 | 0.30 | 0.21 |
| 5.20 | 273.1123 | 2’,6’-OH-4’-OMe DHC [M+H]^+^ | 3.52 | 0.20 | -0.09 |
| 4.79 | 259.0967 | 2’,4’,6’-OH DHC [M+H]^+^ | 3.20 | 0.19 | -0.02 |
| 5.16 | 271.0968 | 2',6'-dihydroxy-4'-methoxychalcone [M+H]^+^ (*) | 3.19 | 0.19 | -0.02 |
| 4.67 | 289.1069 | 2’,6’,4-OH-4’-OMe DHC [M+H]^+^ | 2.50 | 0.15 | 0.04 |
| 4.74 | 121.0649 | Unknown | 2.46 | 0.15 | 0.06 |
| 5.52 | 285.1125 | Unknown | 2.37 | 0.13 | -0.06 |
| 3.60 | 441.1758 | Unknown | 2.07 | 0.11 | -0.04 |
| 4.64 | 221.1175 | Unknown | 2.03 | 0.11 | -0.10 |
| 5.14 | 303.1227 | 2’,6’-OH-4,4’-Me DHC [M+H]^+^ | 1.89 | 0.11 | -0.01 |
| 3.61 | 441.1750 | Unknown | 1.87 | 0.09 | -0.09 |
| 4.24 | 267.0864 | Unknown | 1.75 | -0.10 | -0.14 |
| 4.55 | 205.1952 | Unknown | 1.73 | 0.10 | -0.03 |
|  | | |  |  |  |

**Supplemental Table S2. Variable importance in projection (VIP) scores for mass features detected in whole leaf buds.** Pseudomolecular ion annotations for mass features with VIP score ≥1 based on component 1 of PLS-DA analysis in whole leaf bud extracts. When available, the mass features are annotated based on matching *m/z* and retention time (RT) of analytical standards, including 2’,4’,6’-OH-4-OMe DHC (2’,4’,6’-trihydroxy-4-methoxydihydrochalcone), 2’,6’-OH-4’-OMe DHC (2’,6’-dihydroxy-4’-methoxydihydrochalcone), 2’,4’,6’-OH DHC (2’,4’,6’-trihydroxydihydrochalcone), 2’,6’,4-OH-4’-OMe DHC (asebogenin), and 2’,6’-OH-4,4’-Me DHC (2’,6’-dihydroxy-4,4’-dimethoxydihydrochalcone). Putatively identified mass features are indicated by (*).

| **RT** | ***m/z*** | **Pseudomolecular Ion Annotation** | **VIP score** | **Reference** |
| --- | --- | --- | --- | --- |
| 4.74 | 289.1070 | 2’,4’,6’-OH-4-OMe DHC [M+H]^+^ | 4.99 | English et al., 1991 |
| 4.80 | 285.0762 | 3,7-dihydroxy-5-methoxyflavonol [M+H]^+^ (*) | 4.66 | Greenaway et al., 1990 |
| 4.72 | 287.0915 | 2’,4’,6’-trihydroxy-4-methoxychalcone [M+H]^+^ (*) | 4.60 | English et al., 1991 |
| 5.20 | 273.1123 | 2’,6’-OH-4’-OMe DHC [M+H]^+^ | 3.55 | English et al., 1991 |
| 4.79 | 259.0967 | 2’,4’,6’-OH DHC [M+H]^+^ | 3.23 | English et al., 1991 |
| 5.16 | 271.0968 | 2’,6’-dihydroxy-4’-methoxychalcone [M+H]^+^ (*) | 3.22 | English et al., 1991 |
| 4.67 | 289.1069 | 2’,6’,4-OH-4’-OMe DHC [M+H]^+^ | 2.51 | English et al., 1991 |
| 4.74 | 121.0649 | Unknown | 2.48 |  |
| 5.52 | 285.1125 | Unknown | 2.39 |  |
| 3.60 | 441.1758 | Unknown | 2.08 |  |
| 4.64 | 221.1175 | Unknown | 2.03 |  |
| 5.14 | 303.1227 | 2’,6’-OH-4,4’-Me DHC [M+H]^+^ | 1.90 | English et al., 1991 |
| 3.61 | 441.1750 | Unknown | 1.86 |  |
| 4.55 | 205.1952 | Unknown | 1.74 |  |
| 4.24 | 267.0864 | Unknown | 1.74 |  |
| 3.60 | 325.0922 | Unknown | 1.71 |  |
| 4.95 | 277.2165 | Unknown | 1.71 |  |
| 4.56 | 123.1168 | Unknown | 1.70 |  |
| 5.75 | 205.1953 | Unknown | 1.67 |  |
| 4.70 | 235.1694 | Unknown | 1.66 |  |
| 5.53 | 379.1546 | Unknown | 1.58 |  |
| 4.86 | 301.0709 | 3,5,4’-trihydroxy-7-methoxyflavonol [M+H]^+^ (*) | 1.57 | Greenaway et al. 1992 |
| 3.61 | 325.0917 | Unknown | 1.56 |  |
| 5.52 | 506.1965 | Unknown | 1.54 |  |
| 3.49 | 213.0910 | Benzyl benzoate [M+H]^+^ (*) | 1.53 | English et al., 1991 |
| 4.75 | 257.0811 | 2’,4’,6’-trihydroxychalcone [M+H]^+^ (*) | 1.53 | Greenaway et al. 1992 |
| 5.40 | 403.1545 | Unknown | 1.52 |  |
| 5.41 | 299.0917 | Unknown | 1.50 |  |
| 3.50 | 442.1713 | Salicortin [M+H]^+^ (*) | 1.43 | Boeckler et al. 2011 |
| 5.43 | 283.0969 | Unknown | 1.41 |  |
| 4.99 | 697.3748 | Unknown | 1.39 |  |
| 4.56 | 135.1169 | Unknown | 1.37 |  |
| 4.97 | 421.1653 | Unknown | 1.35 |  |
| 5.01 | 299.0915 | Unknown | 1.31 |  |
| 5.15 | 121.0649 | Unknown | 1.28 |  |
| 3.61 | 163.0390 | Unknown | 1.28 |  |
| 4.66 | 183.0652 | Unknown | 1.27 |  |
| 4.95 | 295.2271 | Unknown | 1.27 |  |
| 4.97 | 419.1492 | Unknown | 1.26 |  |
| 5.43 | 405.1694 | Unknown | 1.24 |  |
| 5.39 | 433.1650 | Unknown | 1.24 |  |
| 5.08 | 419.1494 | Unknown | 1.24 |  |
| 5.49 | 283.0969 | Unknown | 1.24 |  |
| 3.55 | 479.0815 | Quercetin 3-glucuronide [M+H]^+^ (*) | 1.23 | Nissinen et al. 2007 |
| 5.85 | 491.2799 | Unknown | 1.22 |  |
| 4.23 | 546.1970 | Tremulacin [M+NH_4_]^+^ (*) | 1.21 | Boeckler et al. 2011 |
| 5.41 | 313.1072 | Unknown | 1.20 |  |
| 5.19 | 553.2225 | Unknown | 1.17 |  |
| 4.23 | 546.1978 | Tremulacin [M+NH_4_]^+^ (*) | 1.17 | Boeckler et al. 2011 |
| 6.22 | 425.2326 | Unknown | 1.17 |  |
| 5.43 | 311.1277 | Unknown | 1.16 |  |
| 5.10 | 327.1231 | Unknown | 1.16 |  |
| 5.16 | 391.1542 | Unknown | 1.15 |  |
| 5.16 | 167.0340 | Unknown | 1.15 |  |
| 5.16 | 417.1340 | Unknown | 1.12 |  |
| 4.72 | 523.2694 | Unknown | 1.12 |  |
| 2.98 | 135.0443 | Unknown | 1.08 |  |
| 5.02 | 697.3744 | Unknown | 1.08 |  |
| 3.60 | 181.0497 | Caffeic acid [M+H]^+^ (*) | 1.06 | Wang et al. 2017 |
| 4.95 | 335.2196 | Unknown | 1.05 |  |
| 4.76 | 121.0650 | Unknown | 1.03 |  |
| 3.54 | 303.0505 | Quercetin 3-galactoside [M+H]^+^ (*) | 1.02 | Nissinen et al. 2007 |
| 4.29 | 271.0604 | Unknown | 1.01 |  |

**Supplemental Table S3. A shortlist of the variable importance in projection (VIP) scores and loading values for mass features detected in surface resin extracts.** Pseudomolecular ion annotations for the 15 mass features with the highest VIP scores based on PLS-DA analysis of surface resin presented in Fig. 6B for (A) component 1, (B) component 2, and (C) component 3. When available, the mass features are annotated based on matching *m/z* and retention time (RT) of analytical standards, including 2’,4’,6’-OH-4-OMe DHC (2’,4’,6’-trihydroxy-4-methoxydihydrochalcone), 2’,4’,6’-OH DHC (2’,4’,6’-trihydroxydihydrochalcone), and 2’,6’-OH-4’-OMe DHC (2’,6’-dihydroxy-4’-methoxydihydrochalcone). Putatively identified mass features based on accurate mass are indicated by (*). Component 1 explained 36.9 %, component 2 explained 16.1 %, and component 3 explained 16.1 % of the variance in the PLS-DA data model.

**A)**

| **RT** | ***m/z*** | **Pseudomolecular Ion Annotation** | **VIP score** | **Loadings 1** | **Loadings 2** |
| --- | --- | --- | --- | --- | --- |
| 5.21 | 301.1063 | 2’,6’-dihydroxy-4’,4-dimethoxychalcone [M+H]^+^ (*) | 3.99 | -0.23 | -0.14 |
| 4.07 | 277.1767 | Unknown | 3.55 | 0.18 | -0.20 |
| 5.03 | 301.1064 | 5,7-dimethoxy-4'-hydroxyflavanone [M+H]^+^ (*) | 3.52 | -0.19 | 0.15 |
| 5.08 | 121.0645 | Unknown | 3.22 | -0.18 | -0.08 |
| 5.05 | 327.1220 | Unknown | 3.19 | -0.18 | 0.03 |
| 5.40 | 301.1068 | 5-hydroxy-7,4'-dimethoxyflavanone [M+H]^+^ (*) | 3.14 | -0.17 | -0.15 |
| 5.09 | 271.0960 | 2',6'-dihydroxy-4'-methoxychalcone [M+H]^+^ (*) | 3.02 | -0.17 | 0.05 |
| 6.80 | 310.3092 | Unknown | 2.91 | 0.16 | -0.01 |
| 5.33 | 433.1634 | Unknown | 2.75 | -0.15 | -0.11 |
| 5.10 | 421.1644 | Unknown | 2.66 | -0.14 | -0.14 |
| 4.74 | 285.0751 | 3,5-dihydroxy-7-methoxyflavonol [M+H]^+^ (*) | 2.65 | 0.13 | -0.20 |
| 4.06 | 219.1738 | Unknown | 2.64 | 0.13 | -0.12 |
| 5.47 | 553.2224 | Unknown | 2.59 | -0.14 | -0.12 |
| 5.55 | 433.1634 | Unknown | 2.58 | -0.14 | -0.08 |
| 5.35 | 553.2213 | Unknown | 2.52 | -0.14 | 0.04 |

**B)**

| **RT** | ***m/z*** | **Pseudomolecular Ion Annotation** | **VIP score** | **Loadings 1** | **Loadings 2** |
| --- | --- | --- | --- | --- | --- |
| 5.02 | 421.1636 | Unknown | 4.98 | 0.03 | 0.28 |
| 5.21 | 553.2202 | Unknown | 4.39 | 0.02 | 0.24 |
| 4.74 | 285.0751 | 3,5-dihydroxy-7-methoxyflavonol [M+H]^+^ (*) | 3.69 | 0.13 | -0.20 |
| 4.07 | 277.1767 | Unknown | 3.56 | 0.18 | -0.20 |
| 4.66 | 289.1063 | 2',4',6'-OH-4-OMe DHC | 3.27 | 0.18 | -0.20 |
| 4.73 | 259.0958 | 2',4',6'-OH DHC | 2.83 | -0.07 | 0.16 |
| 5.13 | 273.1115 | 2',6'-OH-4'-OMe DHC | 2.80 | -0.04 | 0.17 |
| 5.03 | 301.1064 | 5,7-dimethoxy-4'-hydroxyflavanone [M+H]^+^ (*) | 2.75 | -0.19 | 0.15 |
| 5.40 | 301.1068 | 5-hydroxy-7,4'-dimethoxyflavanone [M+H]^+^ (*) | 2.74 | -0.17 | -0.15 |
| 4.60 | 123.0437 | Unknown | 2.65 | 0.09 | -0.16 |
| 5.10 | 421.1644 | Unknown | 2.58 | -0.14 | -0.14 |
| 5.21 | 301.1063 | 2’,6’-dihydroxy-4’,4-dimethoxychalcone [M+H]^+^ (*) | 2.55 | -0.23 | -0.14 |
| 5.08 | 391.1535 | Unknown | 2.39 | 0.00 | 0.13 |
| 4.73 | 287.0906 | 2’,4’,6'-trihydroxy-4-methoxychalcone [M+H]^+^ (*) | 2.28 | 0.00 | -0.13 |
| 5.47 | 553.2224 | Unknown | 2.274 | -0.14 | -0.12 |

**C)**

| **RT** | ***m/z*** | **Pseudomolecular Ion Annotation** | **VIP score** | **Loadings 1** | **Loadings 3** |
| --- | --- | --- | --- | --- | --- |
| 5.02 | 421.1636 | Unknown | 4.12 | 0.03 | -0.14 |
| 4.66 | 289.1063 | 2’,4’,6’-OH-4-OMe DHC [M+H]^+^ | 3.92 | 0.03 | -0.21 |
| 5.21 | 553.2202 | Unknown | 3.57 | 0.02 | -0.10 |
| 4.07 | 277.1767 | Unknown | 3.02 | 0.18 | -0.14 |
| 4.74 | 285.0751 | 3,5-dihydroxy-7-methoxyflavone [M+H]^+^ (*) | 2.98 | 0.13 | -0.08 |
| 5.13 | 273.1115 | 2',6'-OH-4'-OMe DHC [M+H]^+^ | 2.78 | -0.04 | -0.17 |
| 6.06 | 280.2626 | Unknown | 2.78 | 0.15 | 0.24 |
| 5.03 | 301.1064 | 5,7-dimethoxy-4'-hydroxyflavanone [M+H]^+^ (*) | 2.72 | -0.19 | -0.11 |
| 4.60 | 123.0437 | Benzoic acid [M+H]^+^ (*) | 2.63 | 0.09 | -0.19 |
| 5.18 | 337.1039 | Unknown | 2.59 | -0.05 | 0.14 |
| 5.92 | 254.2473 | Unknown | 2.58 | 0.12 | 0.23 |
| 4.73 | 259.0958 | 2’,4',6’-OH DHC [M+H]^+^ | 2.56 | -0.07 | -0.14 |
| 5.61 | 365.1353 | Unknown | 2.31 | -0.03 | 0.12 |
| 4.93 | 301.1063 | 2’,6’-dihydroxy-4’,4-dimethoxychalcone [M+H]^+^ (*) | 2.29 | 0.04 | -0.10 |
| 6.06 | 263.2361 | Unknown | 2.25 | 0.08 | 0.19 |

**Supplemental Table S4. Variable importance in projection (VIP) scores for mass features detected in surface resin extracts.** Pseudomolecular ion annotations for mass features with VIP score ≥1 based on component 1 of PLS-DA analysis in whole leaf bud extracts. When available, the mass features are annotated based on matching *m/z* and retention time (RT) of analytical standards, including 2’,4’,6’-OH-4-OMe DHC (2’,4’,6’-trihydroxy-4-methoxydihydrochalcone), 2’,6’-OH-4’-OMe DHC (2’,6’-dihydroxy-4’-methoxydihydrochalcone), 2’,4’,6’-OH DHC (2’,4’,6’-trihydroxydihydrochalcone), 2’,6’,4-OH-4’-OMe DHC (asebogenin), and 2’,6’-OH-4,4’-Me DHC (2’,6’-dihydroxy-4,4’-dimethoxydihydrochalcone). Putatively identified mass features are indicated by (*).

| **RT** | ***m/z*** | **Pseudomolecular Ion Annotation** | **VIP score** | **Reference** |  |
| --- | --- | --- | --- | --- | --- |
| 5.21 | 301.1063 | 2’,6’-dihydroxy-4’,4-dimethoxychalcone [M+H]^+^ (*) | 3.99 | Greenaway et al., 1991 | |
| 4.07 | 277.1767 | Unknown | 3.55 |  | |
| 5.03 | 301.1064 | 5,7-dimethoxy-4’-hydroxyflavanone [M+H]^+^ (*) | 3.52 | Greenaway et al., 1991 | |
| 5.08 | 121.0645 | Unknown | 3.22 |  | |
| 5.05 | 327.1220 | Unknown | 3.19 |  | |
| 5.40 | 301.1068 | 5-hydroxy-7,4’-dimethoxyflavanone [M+H]^+^ (*) | 3.14 | Greenaway et al., 1991 | |
| 5.09 | 271.0960 | 2’,6’-dihydroxy-4’-methoxychalcone [M+H]^+^ (*) | 3.02 | English et al., 1991 | |
| 6.80 | 310.3092 | Unknown | 2.91 |  | |
| 5.33 | 433.1634 | Unknown | 2.75 |  | |
| 5.10 | 421.1644 | Unknown | 2.66 |  | |
| 4.74 | 285.0751 | 3,5-dihydroxy-7-methoxyflavonol [M+H]^+^ (*) | 2.65 | Greenaway et al., 1990 | |
| 4.06 | 219.1738 | Unknown | 2.64 |  | |
| 5.47 | 553.2224 | Unknown | 2.59 |  | |
| 5.55 | 433.1634 | Unknown | 2.58 |  | |
| 5.35 | 553.2213 | Unknown | 2.52 |  | |
| 5.42 | 285.1115 | Unknown | 2.43 |  | |
| 6.06 | 280.2626 | Unknown | 2.43 |  | |
| 4.49 | 123.1165 | Unknown | 2.26 |  | |
| 4.49 | 205.1944 | Unknown | 2.26 |  | |
| 5.25 | 133.0645 | Cinnamaldehyde [M+H]^+^ (*) | 2.23 | - | |
| 4.49 | 135.1165 | Unknown | 2.23 |  | |
| 4.06 | 237.1843 | Unknown | 2.19 |  | |
| 4.60 | 183.0648 | Unknown | 2.10 |  | |
| 4.63 | 301.1063 | 5,7-dimethoxy-4’-hydroxyflavanone [M+H]^+^ (*) | 2.10 | English et al., 1991 | |
| 4.82 | 301.0701 | 3,5,4’-trihydroxy-7-methoxyflavonol [M+H]^+^ (*) | 2.02 | Greenaway et al., 1992 | |
| 4.60 | 123.0437 | Benzoic acid [M+H]^+^ (*) | 1.99 | English et al., 1991 | |
| 5.03 | 203.1062 | Unknown | 1.89 |  | |
| 4.59 | 221.1165 | Unknown | 1.87 |  | |
| 5.92 | 254.2473 | Unknown | 1.81 |  | |
| 4.63 | 437.1583 | Unknown | 1.80 |  | |
| 5.10 | 297.1116 | Cinnamyl-caffeate [M+H]^+^ (*) | 1.72 | Greenaway et al., 1990 | |
| 3.65 | 245.0800 | Unknown | 1.72 |  | |
| 4.01 | 301.1038 | Unknown | 1.70 |  | |
| 4.06 | 201.1633 | Unknown | 1.65 |  | |
| 4.90 | 335.2187 | Unknown | 1.64 |  | |
| 6.44 | 270.2783 | Unknown | 1.62 |  | |
| 4.65 | 287.0908 | 2’,6’,4-trihydroxy-4’-methoxychalcone [M+H]^+^ | 1.62 | English et al., 1991 | |
| 5.53 | 211.0751 | Unknown | 1.61 |  | |
| 5.28 | 271.0961 | 2’,6’-dihydroxy-4’-methoxychalcone [M+H]^+^ (*) | 1.60 | English et al., 1991 | |
| 5.11 | 135.0437 | Unknown | 1.59 |  | |
| 5.43 | 299.0913 | Unknown | 1.58 |  | |
| 5.37 | 315.1223 | Unknown | 1.56 |  | |
| 4.49 | 149.1321 | Unknown | 1.55 |  | |
| 3.66 | 275.0905 | Unknown | 1.53 |  | |
| 4.91 | 327.1219 | Unknown | 1.49 |  | |
| 5.73 | 285.1116 | Unknown | 1.47 |  | |
| 4.06 | 159.1163 | Unknown | 1.46 |  | |
| 3.76 | 447.1618 | Unknown | 1.38 |  | |
| 6.13 | 268.2629 | Unknown | 1.38 |  | |
| 5.37 | 403.1532 | Unknown | 1.37 |  | |
| 3.46 | 442.1695 | Salicortin [M+NH_4_]^+^ (*) | 1.34 | Boeckler et al. 2011 | |
| 5.11 | 449.1587 | Unknown | 1.33 |  | |
| 5.11 | 167.0335 | Unknown | 1.32 |  | |
| 6.37 | 142.1222 | Unknown | 1.31 |  | |
| 5.80 | 228.2317 | Unknown | 1.29 |  | |
| 4.73 | 259.0958 | 2’,4’,6’-OH DHC [M+H]^+^ | 1.20 | English et al., 1991 | |
| 6.55 | 270.2787 | Unknown | 1.19 |  | |
| 5.18 | 337.1039 | Unknown | 1.17 |  | |
| 6.37 | 226.2160 | Unknown | 1.16 |  | |
| 6.06 | 263.2361 | Unknown | 1.15 |  | |
| 3.72 | 447.1616 | Unknown | 1.14 |  | |
| 4.65 | 407.1483 | Unknown | 1.14 |  | |
| 6.37 | 212.2003 | Unknown | 1.11 |  | |
| 5.97 | 242.2470 | Unknown | 1.11 |  | |
| 4.83 | 319.0935 | Cinnamyl caffeate [M+Na]^+^ (*) | 1.11 | Greenaway et al. 1990 | |
| 4.19 | 546.1959 | Tremulacin [M+NH_4_]^+^ (*) | 1.10 | Boeckler et al. 2011 | |
| 6.37 | 240.2316 | Unknown | 1.10 |  | |
| 5.29 | 417.1333 | Unknown | 1.08 |  | |
| 4.75 | 459.1412 | Unknown | 1.06 |  | |
| 6.88 | 467.3327 | Unknown | 1.06 |  | |
| 5.32 | 299.0910 | Unknown | 1.05 |  | |
| 5.42 | 283.0957 | Unknown | 1.05 |  | |
| 5.07 | 299.0911 | Unknown | 1.05 |  | |
| 5.74 | 315.1222 | Unknown | 1.01 |  | |
| 4.65 | 271.0959 | 2’,6’-dihydroxy-4’-methoxychalcone [M+H]^+^ (*) | 1.01 | Greenaway et al. 1991 | |

**Supplemental Table S5. Detailed MZmine2 pre-processing parameters of the metabolomics data for the positive ionization data processing.** Pre-processing steps included (A) mass detection, (B) ADAP chromatogram builder, (C) chromatogram deconvolution, (D) isotopic peak grouper, (E) alignment, and (F) gap filling parameters.

|  | **Whole bud extracts** | **Surface resin extracts** |
| --- | --- | --- |
| **A) Mass detection** |  |  |
| Mass detector | Exact mass | Exact mass |
| Noise level Threshold | 2.00E+06 | 5.00E+04 |
| **B) ADAP Chromatogram Builder** |  |  |
| Min group size in # of scans | 5 | 5 |
| Group intensity threshold | 2.00E+06 | 5.00E+04 |
| Min highest intensity | 6.00E+06 | 1.50E+05 |
| m/z tolerance | 5.0 ppm | 5.0 ppm |
| **C) Chromatogram deconvolution** |  |  |
| Method | Local minimum search | Local minimum search |
| Chromatographic threshold | 95% | 95% |
| Search minimum in RT range | 0.05 min | 0.05 min |
| Minimum relative height | 30% | 30% |
| Minimum absolute height | 2.00E+06 | 3.00E+04 |
| Min ratio of peak top/edge | 1 | 1.5 |
| Peak duration range | 0.00-10.00 min | 0.00-10.00 min |
| **D) Isotopic Peak Grouper** |  |  |
| m/z tolerance | 5.0 ppm | 5.0 ppm |
| Retention time tolerance | 0.5 min | 0.5 min |
| Monotonic shape | Yes | Yes |
| Maximum charge | 1 | 1 |
| Representative isotope | Most intense | Most intense |
| **E) Alignment** |  |  |
| Method | Join Aligner | Join Aligner |
| m/z tolerance | 5.0 ppm | 5.0 ppm |
| Weight for m/z | 20 | 20 |
| Weight for RT | 10 | 10 |
| Retention time tolerance | 0.1 min | 0.1 min |
| **F) Gap filling** |  |  |
| Method | Peak Finder | Peak Finder |
| Intensity tolerance | 5.0% | 5.0% |
| m/z tolerance | 5.0 ppm | 5.0 ppm |
| Retention time tolerance | 0.1 min | 0.1 min |
